# Genetic sex determination assays in 53 mammalian species: Literature analysis and guidelines for reporting standardization

**DOI:** 10.1002/ece3.3707

**Published:** 2017-12-13

**Authors:** Karin Hrovatin, Tanja Kunej

**Affiliations:** ^1^ Department of Animal Science Biotechnical Faculty University of Ljubljana Domzale Slovenia

**Keywords:** genetic sex, mammals, molecular sexing, reporting standardization, sex determination

## Abstract

Erstwhile, sex was determined by observation, which is not always feasible. Nowadays, genetic methods are prevailing due to their accuracy, simplicity, low costs, and time‐efficiency. However, there is no comprehensive review enabling overview and development of the field. The studies are heterogeneous, lacking a standardized reporting strategy. Therefore, our aim was to collect genetic sexing assays for mammals and assemble them in a catalogue with unified terminology. Publications were extracted from online databases using key words such as sexing and molecular. The collected data were supplemented with species and gene IDs and the type of sex‐specific sequence variant (SSSV). We developed a catalogue and graphic presentation of diagnostic tests for molecular sex determination of mammals, based on 58 papers published from 2/1991 to 10/2016. The catalogue consists of five categories: species, genes, SSSVs, methods, and references. Based on the analysis of published literature, we propose minimal requirements for reporting, consisting of: species scientific name and ID, genetic sequence with name and ID, SSSV, methodology, genomic coordinates (e.g., restriction sites, SSSVs), amplification system, and description of detected amplicon and controls. The present study summarizes vast knowledge that has up to now been scattered across databases, representing the first step toward standardization regarding molecular sexing, enabling a better overview of existing tests and facilitating planned designs of novel tests. The project is ongoing; collecting additional publications, optimizing field development, and standardizing data presentation are needed.

1

- Genetic sex determination assays feature high specificity, sensitivity, effectiveness, low time consumption, and adequacy of small samples.
- Current reporting of molecular sexing tests is highly heterogeneous.
- The molecular sexing field could benefit from more clearly defined terminology and standardized reporting form.

## 1 INTRODUCTION

There are many nonmolecular sexing methods, which are nowadays mostly being replaced by assays based on sex‐specific sequence variants **(**SSSVs), which are genetic sequence differences between sexes, for example indels on Y and X orthologous genes (Murata, Ogura, & Kuroiwa, 2011; Sullivan, Mannucci, Kimpton, & Gill, 1993), male‐ or female‐specific genetic dose (Mittnik, Wang, Svoboda, & Krause, 2016), sex‐specific sequence polymorphisms (single‐nucleotide polymorphisms between orthologous genes on X and Y chromosomes) (Statham, Turner, & O'Reilly, 2007), and others described in Figure S1. Genetic methods have the advantage of being less time consuming in certain situations (use of noninvasive samples instead of laborious tracking down of the animal), require smaller samples, are easier to perform, and can be noninvasive (e.g., hair). In the field of embryo sexing, many implant sexing techniques, such as karyotyping (Yano, 1993), are limited by the lack of material, while molecular assays need as little as a single blastomere (Tsai et al., 2011). Measuring the activity of X chromosome‐linked enzymes (Yano, 1993) or RNA‐based PCRs is further complicated by the presence of some gene products only at certain developmental stages (Prantner, Ord, Medvedev, & Gerton, 2016); this problem is not present when the test is based on the DNA. Furthermore, embryo freezing is not needed (contrary to in situ hybridization), reducing the chance of freezing‐induced damage (Mara et al., 2004). Embryos can be sexed prior to the implantation into the host mother opposed to ultrasound and fetal DNA detection in the blood of pregnant females (Tavares et al., 2016). Moreover, the accuracy of still often used ultrasound strongly drops when there are more fetuses (Prugnard et al., 2016), and some new‐born mammals cannot be anatomically sexed (48% of mice) (Clapcote & Roder, 2005).

The information of gender proportions in a wild‐living community is useful as it predicts pregnancy rates for the future and so allows population management. As rare animals are arduous to capture (Statham et al., 2007) and observe (Ortega, Franco, Adams, Ralls, & Maldonado, 2004), it is important to develop sexing techniques that can be performed on highly degraded noninvasive samples such as feces and hair. The same goes for analysis of ancient remains, such as juvenile skeletons that cannot be sexed anatomically (Gibbon, Paximadis, Strkalj, Ruff, & Penny, 2009), or forensic samples. When using such materials, there is higher risk for false negatives and contamination (Pages et al., 2009); therefore, the amplicon should be as short as possible (Statham et al., 2007) (maximally 200–250 bp) (Pages et al., 2009) and species specific (Esteve Codina, Niederstatter, & Parson, 2009). Higher sensitivity and thus detection of low amounts of DNA can be achieved with loop‐mediated isothermal amplification (LAMP) (Lee, 2017). An approach for reducing cross‐contamination is the use of certain sample types that are less prone to containing impurities, for example, the use of muscle tissue instead of hair (Campbell, Pauli, Thomas, & McClean, 2010). Molecular sexing is especially important in rape victim identification as an alternative to spermatozoa detection, which can produce false negatives due to condom use or lack of ejaculation. On the contrary, epithelial cells or leukocytes of the assaulter, which are more prone to be present, can be determined by genetic methods (Campos et al., 2014).

There are many approaches increasing sexing accuracy and sensitivity. When working with nonrepetitive sequences such as genes (for example *SRY* and *ZFY*/*X*) in small samples, nested PCRs are sometimes necessary due to limited amount of DNA (Gutierrez‐Adan, Cushwa, Anderson, & Medrano, 1997). One alternative to PCR is LAMP, which is highly specific, sensitivity, and needs no for further electrophoresis—a white precipitate is formed, that can be detected using turbidimetry (Hirayama et al., 2006). Lately, multiplex PCR reactions have been often used, although the concentrations of PCR mixture components must be heavily guarded. Therefore, the simplex assay possesses an advantage in terms of preparation time and costs (Clapcote & Roder, 2005). The automatization of detection with the use of fluorescently labeled primers has even further eased the sexing process (Sullivan et al., 1993) as well as the visualization of results with capillary techniques reducing potential human mistakes and increasing throughput analysis (Campbell et al., 2010). Development of new tests could be speeded up as already defined assays can be used on additional species due to interspecies homology between sequences, but this potential is currently largely neglected. For example, a test based on the *ZFY*/*ZFX* enables sex determination in four species, human, cattle, goat, and sheep; moreover, the primers used are universal, amplifying across a wide range of species (Aasen & Medrano, 1990). Often, due to the fear of cross‐species contamination, when using conserved sequences such as *SRY* (Gutierrez‐Adan et al., 1997), species‐specific primers are desired. New sexing loci for nonmodel organisms can be detected using next‐generation genome sequencing and programs assigning output sequences to sex chromosomes, as described in Gautier (2014).

One of the most profound challenges of molecular sexing and its accuracy is still the sex bias resulting in false negatives (males determined as females), for example, when the test is based on male‐specific sequence with less genomic copies than internal positive control (IPC) (Baumgardt et al., 2013), especially when using mitochondrial DNA as an IPC (Bidon et al., 2013). This becomes even more troubling if male fragments are longer than female. Approaches to solve these obstacles might be the simultaneous amplification of two Y chromosome‐specific regions and one IPC (Madel, Niederstatter, & Parson, 2016) or use of a repetitive Y‐specific sequence (Benoit, Quatrehomme, Carle, & Pognonec, 2013). However, separate PCR reactions for male and female sequences always pose a risk for false negative due to amplification failure. Thus, multiplex PCR analysis can be applied; even so, amplification of sex nonspecific region cannot prove the effectivity of male‐specific primers—therefore the use of orthologous genes on sex chromosomes (e.g., *ZFX* and *ZFY*), requiring only one set of primers, has been advised in Fontanesi et al. (2008). False negatives can be also produced due to allelic dropout, for example, when using human *AMELY* (Tozzo et al., 2013).

Molecular sexing is a broad, rapidly growing field. However, there is still no consensus among scientists on data presentation and terminology regarding methods and SSSVs. Due to the lack of a systematic review article on this field, the only condensed overview that reader could have gained was from paper introductions that are limited in length and concentrated specifically on one area. Therefore, the main challenges of the field are as follows: (1) complicated information exchange due to incomprehension of ambiguous terms; (2) lack of information in some articles due to unstandardized format; and (3) stunted and not interdependent development of assays, as it is not clear in which direction the development of new methods should proceed due to the shattered nature of the field of molecular sexing.

Therefore, the aim of this was to: (1) collect existing sexing assay data in a tabular format and review the current characteristics of the molecular sex determination field; (2) complement the collected information from published sexing assays (with IDs, scientific names etc.) as well as unifying terminology regarding SSSVs and methods and supplementing them with schematic explanations; and (3) establish minimal requirements for reporting molecular sexing assays as well as some useful guidelines.

## 2 MATERIALS AND METHODS

Published articles were extracted from Web of Science and PubMed. The search was defined by the characters in []: [(sexing or (sex determi*) or (sex identif*) or (gender identif*) or gender (determi*))] and using one or more of specifications: [and mammal*], [and (gen* or molec*)]. Additionally, some of the articles regarding sex determination in humans were extracted from Butler and Li (2014). Time span of publication search defined in Web of Science and PubMed search engines was from 1991 to 06/2017.

The data extracted from publications were ordered in an Excel table (Table S1). When sorting sexing methods for the same species, the most recent ones were described at the bottom (in the table). From published literature, we extracted the following data: species and gene names, methods used, and SSSVs. We added species and gene IDs, scientific species name, unified the SSSV, and method terminology. Species IDs were extracted from National Center for Biotechnology Information (NCBI) (NCBI Resource Coordinators, 2017) Taxonomy browser. Names of human and mouse genes were updated according to the HUGO Gene Nomenclature Committee (HGNC) (Gray, Yates, Seal, Wright, & Bruford, 2015) and Mouse Genome Database (Blake et al., 2017) and others in accordance to NCBI gene nomenclature if available. Gene synonyms used in articles are written in brackets. When only the product of the gene was reported, the gene name was added. Gene IDs were extracted from NCBI gene, if not available, they were replaced with sequence IDs from NCBI nucleotide, if provided in articles. The diagrams were drawn according to the data in articles or general characteristics of the presented method.

## 3 RESULTS

The 58 research articles obtained were published from 1991 to 10/2016, and all of them introduced a new molecular sexing method in mammals. Assays in collected data apply to 53 mammalian species, most of them to humans (22 articles), followed by sheep (five), cattle (four), and mice (four). More detailed information about animal species is available in Table S1. All the collected data with complemented information and unified terminology are presented in Figure S1 and Table S1, which includes 117 molecular sexing assays (identical assays used on multiple animals are counted once for each animal species sexed). For sex‐specific sequence variant, we introduced an abbreviation SSSV and for sex‐specific sequence polymorphism SSSP.

### 3.1 Data extraction from publications and complementation

All extracted data were collected in Table S1 and complemented with additional information about animal species, genomic locations, and sexing methods; all of them described below. The graphic presentation includes all relevant information regarding polymorphisms and assays used in the tests (Figure S1) including polymorphisms type, most often used techniques, expected results, and references. Moreover, details regarding functioning of amplification and detection techniques are explained. There is a table next to each figure summarizing the articles using the same approach.

#### 3.1.1 Species and samples

Sexing assays collected in this study were conducted on field material for ecological research (fecal samples, hair, etc.), archeological remains, meat control, forensic evidence (for example saliva), and assays for laboratory and prenatal diagnostics or embryo transplantation. Animals sexed were both domestic (such as dogs, goats) and wild (various bear species, foxes, beaver, elephants, etc.). Common and scientific species names were included in 27 articles, only common names were available in 25 articles. Six articles did not specify the species name; however, it was possible to extract this information from the context, for example, forensic study, Indian population, and human gene. None of the articles contained species identification number (ID) from NCBI taxonomy browser. The IDs were complemented as stated in methods, for example, 10088 for mouse and 9606 for human.

#### 3.1.2 Genetic loci

Sex determination tests are based on Y‐specific sequences and sex nonspecific sequences on X chromosome, autosomes, and mtDNA. Genes that were most often used in the sexing assays are *SRY*,* AMELY* with its homologue *AMELX* and *ZFY* and homologue *ZFX*. *SRY* was used for sexing humans (Esteve Codina et al., 2009), bears (Pages et al., 2009), cattle (Gokulakrishnan, Kumar, Sharma, Mendiratta, & Sharma, 2012), dogs (Prugnard et al., 2016), mouse (Prantner et al., 2016), sheep (Mara et al., 2004), buffalo (Fu et al., 2007), minks, ermines, badgers, otters and pine martens (Statham et al., 2007), elephants (Gorrell et al., 2012), koalas (Wedrowicz, Karsa, Mosse, & Hogan, 2013), and sea mammals (McHale, Broderick, Ovenden, & Lanyon, 2008). Sex determination was also performed on the following genes: *DDX3Y* and homologue *DDX3X, EIF2S3Y* and homologue *EIF2S3X*,* Jarid1d* and homologue *Jarid1c*,* Sly* and *SMCY* (Table S1). Beside genes, authors also reported the use of markers, for example, *FBNY* and homologue *FBN17* and repeated sequences, such as *BuRY.2* and *BuRYN.I* (Table S1). The preceding sequences are all located on Y chromosome and their homologues on X chromosome, except when stated else how.

To verify the absence of Y‐specific amplification as a female sample, an IPC must be included to confirm that the amplicon is not missing due to amplification reaction or detection failure. Thus, the gender is judged male when both sequences (IPC and Y chromosome‐specific sequence) are successfully amplified, while only male–female common or only male‐specific positive reaction indicates female sex or that no sex can be determined, respectively (Hirayama et al., 2006). IPCs were sequences homologous to Y located on X (for example Morikawa, Yamamoto, & Miyaishi, 2011), autosomes (as in Prantner et al., 2016), or mtDNA (also used by Pages et al., 2009). Such sequences are as follows: *MY‐CYB*,* MYOG*,* Il2*,* G3PDH*,* G6PD*,* Xlr, ACTB*,* DXZ4*,* STS*,* PLP*,* GAPDH* gene, 12S and 16S rRNA, 12S mtDNA, 1.715 satellite, and Alu sequences. Lately, the developers of the tests strive to shorter amplicons that enable correct identification even in degraded samples (Tschentscher, Frey, & Bajanowski, 2008).

Incorrect terminology or old gene names were used in 18 of 58 articles, using incorrect nomenclature (e.g., lack of capital letters) or only gene product (protein) names; therefore, this information was updated or supplemented in this study. Ten articles included sequence names that could have not been found in NCBI database either due to lack of information in the article or the sequence apparently not being in the database. Therefore, gene names often could not be updated according to the HGNC nomenclature, as articles did not contain exact gene names, but rather only abbreviations not found on NCBI, poorly defined names, and product (protein or ribosomal RNA) names. Information about products was sometimes not enough to find appropriate gene as there were more sequences related to the protein. All the sequence IDs are cited in 25 articles and seven articles contain only some sequence IDs. Some of those articles, when using multiple genomic locations for sexing, reported the gene IDs for only certain sequences. We were not able to complement some sequence IDs, either due to lack of information in the article or due to the fact that they have not yet been deposited in the NCBI database.

#### 3.1.3 Sex‐specific sequence variants

**Figure 1:**
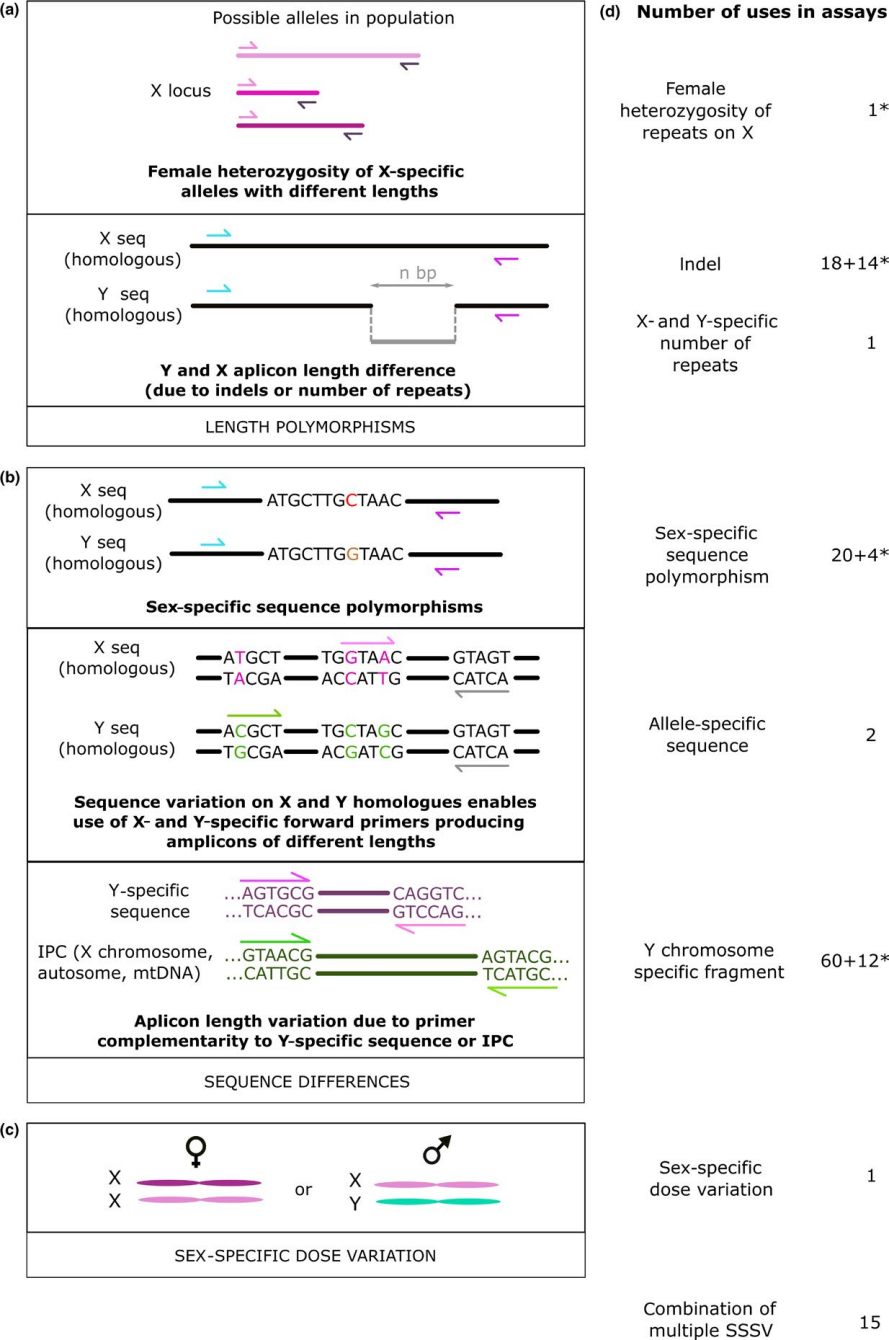
Sex‐specific sequence variants (SSSVs) used for sexing mammals. (a). Length polymorphisms; upper: female heterozygosity of repeats on X; lower: indel and X‐ and Y‐specific number of repeats. (b). Sequence differences; upper: SSSP; middle: allele‐specific sequences; lower: Y chromosome‐specific fragment. (c). Sex‐specific dose of sex chromosomes. (d) Categories of SSSVs used in published reports. Certain studies proposed multiple sexing assays, thus each assay is counted individually. Identical assays used on multiple animals are counted once for each animal species sexed. Certain assays relied on multiple SSSVs, which are thus counted separately (*) and collectively below (number of all assays using multiple SSSVs). *Assays with multiple SSSVs. IPC, internal positive control; seq, sequence; mtDNA, mitochondrial DNA

Sex‐specific sequence variants (SSSVs) used to distinguish between sexes can be ordered in three main types (Figures 1 and S1): (1) length polymorphisms, (2) sequence differences, and (3) number (dose) of sex chromosome (e.g., male one X and one Y, female two X). Length variation arises due to indels on Y and X homologous genes, number of repeats on X chromosome (female heterozygosis), and X‐ and Y‐specific number of repeats. Sequence differences include Y chromosome‐specific fragments (with no homologous gene on X chromosome) and polymorphisms on homologous sex chromosomes; those are allele‐specific sequences (parts of homologous genes that differ in sequence) and sex‐specific sequence polymorphisms (SSSP; single‐nucleotide variations on homologous genes on both sex chromosomes). Certain assays relied on multiple genes and thus more than one SSSV. Furthermore, high‐resolution melting curve (HRM) variation between amplicons is a combined result of various polymorphisms on homologous sequences. Melting point is a temperature at which half of dsDNA dissociates in ssDNA; this can be measured with the use of fluorescent dies. The oldest techniques were based on detection of Y chromosome‐specific fragments (in Akane et al. (1991)) and indels (in Akane et al. (1991) and Sullivan et al. (1993)). Assays based on both SSSVs are still in use today, such as in Prugnard et al. (2016) and Tavares et al. (2016). Overall use of SSSVs in assays presented in the reviewed studies for the purpose of sexing is presented in Figure 1.

#### 3.1.4 Amplification

The amplification of sample DNA to the required quantity is achieved by simplex and multiplex (including duplex) PCR, nested PCR, qPCR, LAMP reaction, and early stages of pyrosequencing (Figure S1). A lot of the authors used PCR variations that either enhance specificity and detection limits (nested PCR) or ease the procedure (simplex and multiplex PCR). When using LAMP, no further detection technique is needed, as the sole positive reaction yields white precipitate. The same goes for qPCR, where the measured fluorescence indicates dose variation (copy number) or presence of specific Y sequence that has been amplified.

#### 3.1.5 Detection

After PCR techniques, further separation and detection of the products are needed (Figure S1). This can be performed using gel or capillary electrophoresis followed by sex determination based on length variation due to indels, restriction (present SSSPs), sex‐specific number of repeats, X alleles length polymorphisms leading to female heterozygosis, and Y‐specific sequences that differ from IPC enabling specific primer positioning leading to amplicon length variation. Lower amounts of the sample DNA can be detected using repetitive sequences or microsatellites (present in intergenic sequences) and genes with higher copy number (on mitochondrial genome), thus gaining more amplicons. For higher certainty of sex determination, another positive control may be included (second male‐specific sequence). Similarly, different SSSVs may be investigated using multiple genes in one assay—Y‐specific sequence, IPC on X chromosome whose homologue on Y chromosome contains an indel, heterozygosity of X loci etc. When doing this in multiplex, amplicons must be selected with an appropriate length difference (not too short to be detected or too long to be run on the same gel).

Amplified fragments can be also analyzed with pyrosequencing or Sanger sequencing and melting curve analysis. The presence of slightly different alleles on X and Y chromosome with single‐base differences (SSSPs) or indels leads to double peaks when using Sanger sequencing. Melting points vary due to different lengths and GC to AT ratios of sex‐specific amplicons; the effect can be enhanced by primers with noncomplementary 5′ tails that have different melting characteristics. A special approach is fragmentation of whole genome followed by amplification and shotgun sequencing. The number of reads aligning to each chromosome determines the copy number, which is, when regarding X and Y chromosomes, specific for a gender.

### 3.2 Minimal requirements for reporting genetic sex determination

Our review and analysis revealed that several articles are missing various types of relevant information, most often gene IDs and sufficient method and data presentation, for example, lack of tables, figures, and clear straight forward explanations. Nucleotide sequences used are often loosely characterized as follows: Gene names are not in accordance with NCBI and HGNC nomenclatures, only products and not gene names are reported. In some cases, when reporting test based on intergenic sequences that do not possess characteristic names, the genome location that would define the sequence is absent. The terminology is highly heterogeneous and hard to unify due to lack of a term database. Therefore, we here suggested some guidelines for reporting sexing assays in scientific literature. The minimal requirements for reporting genetic sex determination (Table 1) and some suggestions are written in the following chapters. Each requirement in the Table 1 is accompanied with additional explanation and recommended article section, where the subject should be dealt with. Topics, where the required place is »abstract« should be there only summarized and then further explained in the body of the paper.

**Table 1:** Minimal requirements for reporting molecular sexing assays

Minimal requirements	Additional explanation, source databases	Section in the article	Example
Species scientific name	NCBI Taxonomy, https://www.ncbi.nlm.nih.gov/taxonomy	Abstract, methods	*Homo sapiens*
Species ID	From NCBI Taxonomy, https://www.ncbi.nlm.nih.gov/taxonomy	Abstract, methods	9606
Name of gene/genetic sequence for sexing	NCBI	Abstract, methods	*SRY* gene
Gene or genetic sequence ID	NCBI acc. No or Gene ID, https://www.ncbi.nlm.nih.gov/gene/, https://www.ncbi.nlm.nih.gov/nuccore/	Abstract, methods	6736, GenBank: JQ811934.1
Sex‐specific variant	/	Abstract, methods	Y chromosome‐specific segment, indel, SSSP
Sexing method	Name and key components of the assays, methodology, amplification, and detection method	Abstract, methods	Nested PCR, multiplex PCR, LAMP, gel electrophoresis, Sanger sequencing
Nucleotide sequence of the region used for sexing		Methods or results	Male: 5′ GTTGACGFemale: 5′ GTCGACG
Locations of important regions on the nucleotide sequence	Presented on the nucleotide sequence; for example, primer alignment, restriction site, SSSV	Methods	Male: 5′ GT**T**GACGFemale: 5′ GT GACGBold: indel; underlined: primer annealing
Characteristics defining the amplicon system	Best to be presented in a table; primers, genetic sequence name, species name (if using more species), amplicon length in each sex/other sex‐determining characteristics, type of PCR (if used) etc.	Methods	Primers: forward 5′AT…, reverse 5′ GT…Gene and amplicon: AMELY (96 bp) and AMELX (90 bp)Female: 1 band (90 bp); male: 2 bands (90 and 96 bp)
If using nested PCR: characterization of inner and outer primers and their products		Methods	Inner primers: forward 5′ GT…, reverse 5′ AT…; product: 60 bpOuter primers: forward 5′ CT…, reverse 5′ AC…; product: 91 bp
Pyrosequencing: dispensation order		Methods	ATCGATCG
Length and identity of each gel band/electrophoreogram peak	Presented on results figure near each band/peak	Results	On figure: “SRY, 76 bp”
Positive and negative controls	On figure/in table alongside other results	Results	On figure: “male positive control”
Reference PMID or WoS ID	In review articles	Results	PMID: 189765

ID, identification number; LAMP, loop‐mediated isothermal amplification; PCR, polymerase chain reaction; SSSP, sex‐specific sequence polymorphism; SSSV, sex‐specific sequence variant.

#### 3.2.1 Abstract information

Abstract section should include the following relevant information: species ID (according to the NCBI database) and its scientific name, clearly stated name of the gene/sequence used for sexing with its ID (from NCBI) and SSSV, named according to the terminology and the method.

#### 3.2.2 Method description

The description of the method should contain nucleotide sequence and the genomic location of primer alignment or other parts important for the assay (SSSV, restriction site etc.). An example of a figure containing the relevant information is in Gokulakrishnan et al. (2012). Furthermore, systematically ordered characteristics for each amplicon or system must be included, best to be presented in a tabular format. Such a table should contain PCR primers (sequence, length), name of the gene or species (when sexing multiple species), amplicon length in each sex/other sex identification characteristics, stated if it is a multiplex or simplex PCR and other important information regarding a specific system. Authors need to be more precise about the naming the methodology, for example, quantitative PCR (qPCR) is often named as real‐time PCR (RT‐PCR), which can be confused with reverse transcription PCR (also abbreviated RT‐PCR) (Bustin et al., 2009). When using nested PCR, the primers and products of inner and outer reaction should be clearly marked. A good example for nested PCR system reporting is in Luptakova et al. (2011). The dispensation order of pyrosequencing reaction should be included as only then the expected pyrogram can be constructed; as presented in Tschentscher et al. (2008). A graphical visualization with expected results for each gender is recommended. An example of a clear and transparent presentation of results that includes both table and the scheme was reported by Pages et al. (2009). When presenting a method, that is less commonly used or more complex, a graphic presentation of the procedure is a welcome addition. For example, the representation of embryo sexing workflow in Tavares et al. (2016).

#### 3.2.3 Results presentation

When presenting the results on gel electrophoresis or as an electrophoreogram, the length and amplicon identity (e.g., IPC, Y‐specific amplicon) should be written near each band/peak position; gender symbols near every line greatly contribute to the visualization; for example, as clearly presented in Weikard et al. (2001). If done so, the reader does not need to search for the information in often densely packed picture description. Positive and negative controls must be clearly presented as it was in Morikawa et al. (2011). Review articles should include PubMed ID (PMID) or Web of science ID (WOS ID) of cited papers.

#### 3.2.4 Validation of the method (results)

Test efficiency (samples that resulted in positive PCR amplification), accuracy (correctly typed samples), and sensitivity (minimal amount of needed DNA/cells) or of other test results can be summarized in one table to assure greater overview. An example of such a clear overview was presented in Mara et al. (2004). This enables the reader to compare different sexing assays without laborious search for relevant information in the text, especially when there are multiple validation tests in one article.

In general, presentation is schemes and tables greatly simplify quick comprehension of the described assay—in some articles, the general overview of the assay was gained only after reading whole methods and results sections.

## 4 DISCUSSION

In the present study, we systematically collected in a tabular form the key information of various sexing assays and complemented the missing information, when possible. For easier understanding, the methods and SSSVs used in molecular sex identification are graphically presented. According to the most common and troubling deficiencies, we established minimal requirements for future sexing assay reporting.

The unified nomenclature and reporting standards would contribute to organizing and developing of the molecular sexing field, but further progress regarding assays planning is also needed. This covers (1) greater attention to interspecies homology and (2) new gene/sequence candidates for sexing. Firstly, there are many sexing assays that have already been used for species other than they were designed for (for example, buffalo [*Bubalus bubalis*] instead of cattle [*Bos taurus*]), and often only minor primer modifications are needed for higher amplification efficiency (Appa Rao & Totey, 1999). Many genes are highly conserved through mammal species (e.g., *ZFY* and *ZFX*); therefore, a study examining which already developed tests could be used for sexing other animals would save time used for laboratory work. So, only minor adjustments and validation of the assays would be needed. This benefit could be maximized by intentional development of tests that are applicable to various species (Aasen & Medrano, 1990; Bidon et al., 2013; Ortega et al., 2004).

Secondly, many assays are currently based only on few genes, for example, human *SRY*, which has already been proven as problematic due to allelic dropout. Therefore, a systematic in silico screening proposing new gene candidates based on SSSVs for test designing is needed. Such collection would enable quick and simple assay development as well as provide a means for more variation in the sexing field. Namely, many authors struggle with the efficient use of genomic tools for gene selection or do not realize their potential. Therefore, they rely only on already well‐characterized sequences and their SSSVs; for example, a 6‐bp deletion in human *AMELX* homologues to *AMELY* (Steinlechner, Berger, Niederstatter, & Parson, 2002; Sullivan et al., 1993).

Our research group has recently focused on reporting standardization in various genomics subfields. We have proposed several initiatives and minimal requirements, for example, for reporting methodologies, such as NGS (Pipan & Kunej, 2015), genotype–phenotype associations (Traven, Ogrinc, & Kunej, 2017; Urh & Kunej, 2016), and interactomics; transcription factor–target interaction (Slemc & Kunej, 2016) and microRNA–target interactions (Piletic & Kunej, 2017). However, there has been no initiative for reporting standardization on the molecular sexing field, although there is great need for standards that would enable faster and more directed development of the field.

## 5 CONCLUSION

Molecular sexing assays are among the most reliable methods, providing results even for samples that cannot be sexed anatomically (for example saliva). Lately, the ease of their use and the time needed have also improved. However, efforts to group and systematically order both terminology and practical experience with corresponding assays have been minimal. Therefore, the growing field clearly needs more organizations, on one hand to avoid confusion regarding the naming of methods and polymorphisms and on the other hand to ease further development of assays. This article presents the first step toward standardization and presents a direction for further development of the field of molecular sexing. In the future, it should also be considered for which species there is a need for further development of assays and which existing tests could be used for other species due to homology.

## CONFLICT OF INTEREST

None declared.

## AUTHOR CONTRIBUTIONS

Karin Hrovatin collected and analyzed the data, drafted the manuscript, and drew the figures. Tanja Kunej designed and coordinated the study and edited the final version of the text.

## DATA ACCESSIBILITY

All the data are included in the manuscript and Supporting Information. Assay catalog: online supporting information, Table S1.
