# Supplementary material for: Genetic sex determination assays in 53 mammalian species: Literature analysis and guidelines for reporting standardization
Source: Ecol Evol. 2017 Dec 13;8(2):1009–18. doi: 10.1002/ece3.3707 (PMC5773321; doi:10.1002/ece3.3707)
Supplement: Supplementary file 1 [file ECE3-8-1009-s001.pdf]

Fig. S1. Polymorphisms with amplification and detection techniques used in articles.

Index

Amplification techniques 2

Simplex, duplex and multiplex PCR 3

Nested PCR 4

qPCR amlification of Y specific and IPC sequence with flourescent primers 5

qPCR amlification of Y specific and IPC sequence with flourescent probe 6

LAMP reaction of Y (male) specific and IPC region... 7

Electrophoreses 8

Sex specific sequence variants 9

Length polymorphisms 10

Separation of different length fragments on the gel due to an indel in X and Y specific gene 11

Detection of PCR products with capillary electrophoresis.................................... 12

Separation of different length fragments on the gel due to specific number of repeats in X and Y specific regions 13

Female heterozigosity of X chromosome loci 14

Sequencing & double peaks (due to indel and thus sequencing frame shift) identification.. 15

Sequence differences 16

Amplification of Y specific and internal positive control (IPC) followed by gel electrophoresis; length variation due to placing of sequence specific primers.... 17

Amplification of two Y specific and one internal positive control (IPC) followed by gel electrophoresis; length variation due to placing of sequence specific primers 18

Sex chromosome alelle specific amplification; length variation due to placing of sequence specific primers - ARMS 19

PCR-RFLP analysis of sex specific sequence polymorphism 20

Sequencing & double peaks (due to SSSP) identification 21

Pyrosequencing & detection of higher/lower/absent peaks due to SSSPs and indels in X and Y homologous sequences... 22

Other polymorphisms............................. 23

Shotgun sequencing and calculation of Ry and Rx from number of alignments to sex chromosomes due to sex specific dose variation.. 24

Analysis of a sex specific melting curve; Y specific sequence and IPC.................... 25

Amplification techniques

- PCR
- qPCR
- LAMP

Simplex, duplex and multiplex PCR

Method	Simplex and duplex PCR
Sex sequence variant	Indel, SSSP, Y specific sequence + IPC
Detection	Electrophoresis
References for simplex PCR	Weikard et al., 2001; Clapcote and Roder, 2005; Fu et al., 2007; Morikawa et al., 2011; McFarlane et al., 2013; Pratner et al., 2016
References for duplex PCR	Tozzo et al., Yano, 1993; Rao and Totey 1999; Mara et al., 2004; Pages et al., 2009; Katsushima et al., 2010; Gokulakrishna et al., 2012; Gorrell et al., 2012; Kamodoyova et al., 2013; Korstian et al., 2013;Salabi et al., 2014; Prantner et al., 2016
References for multiplex PCR	Sullivan et al. 1993; Steinlechner et al., 2002; Mchale et al., 2008; Codina et al., 2009; Sekiquchi et al., 2010; Ahlering et al., 2011; Morikawa et al., 2011; Wedrowicz et al., 2013; Jacot et al., 2013; Madel et al., 2016

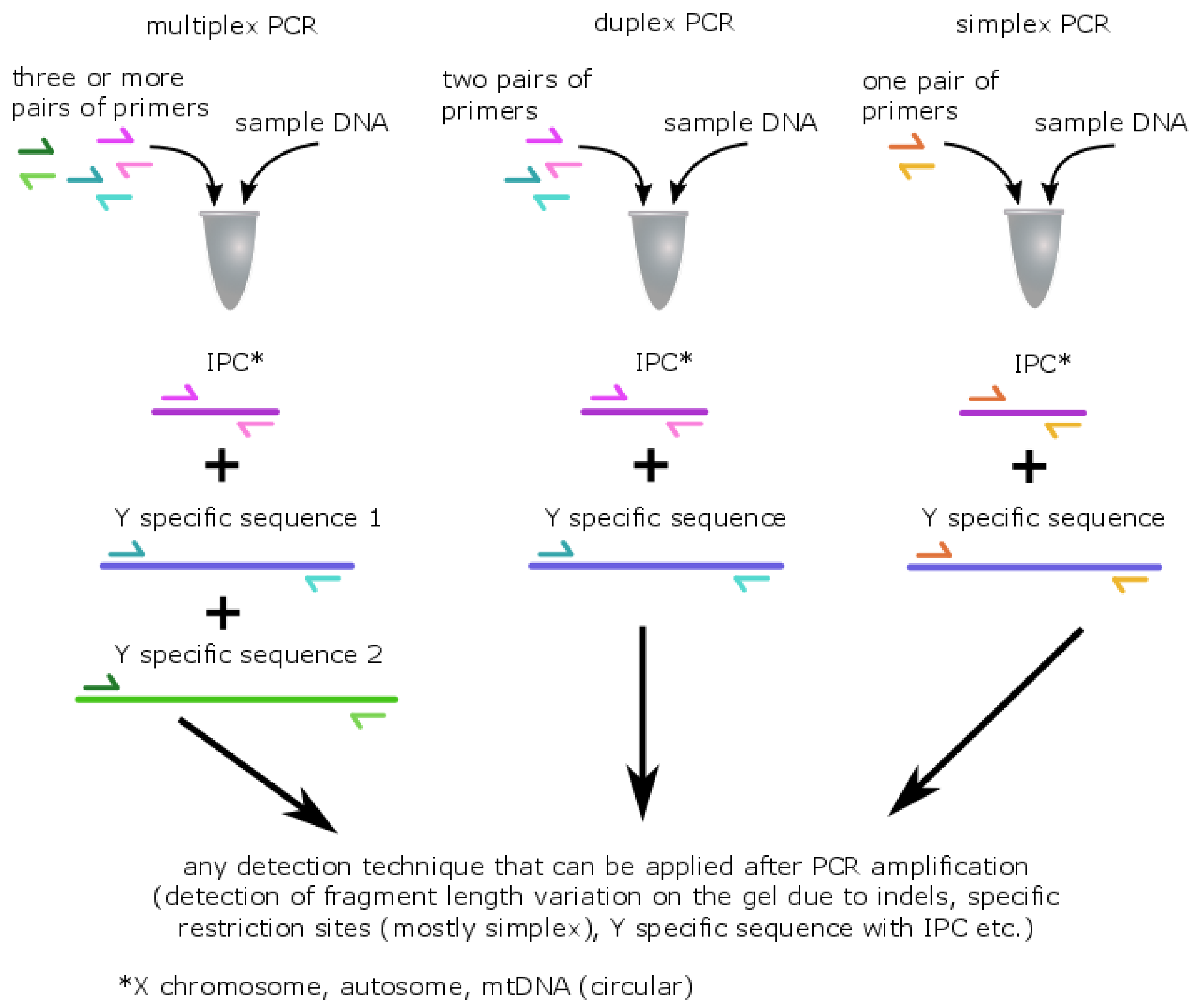

PCR – polymerase chain reaction; IPC – internal positive control; mtDNA – mitochondrial DNA; SSSP – sex specific sequence polymorphism

Nested PCR

Method	Nested PCR
Sex sequence variant	SSSP, indel, Y specific sequence + IPC
Detection	Electrophoresis
References	Gutiérrez-Adán et al., 1997; Rao and Totey, 1999; Fu et al., 2007; Gibbon et al., 2009; Sekiquchi et al., 2010; Luptakova et al., 2011; Camposet al., 2014

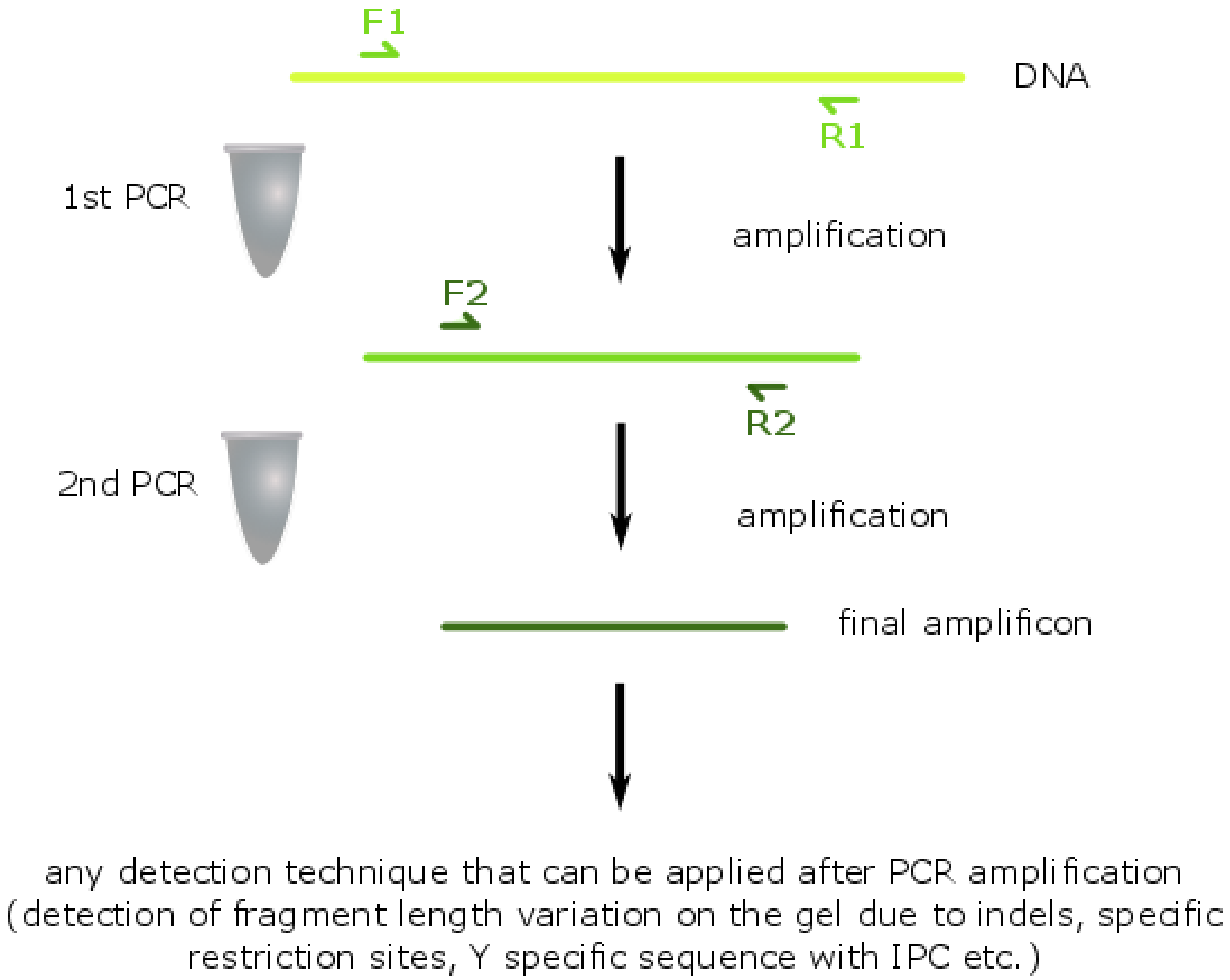

PCR – polymerase chain reaction; IPC – internal positive control; SSSP – sex specific sequence polymorphism

qPCR amlification of Y specific and IPC sequence with flourecent primers

Method	qPCR
Sex sequence variant	Y specific sequence + IPC
Detection	qPCR – flourescence of digested primers
References	Zimmermann et al., 2005; Blagodatskikh et al., 2010; Benoit et al., 2013; Kamodoyova et al., 2013

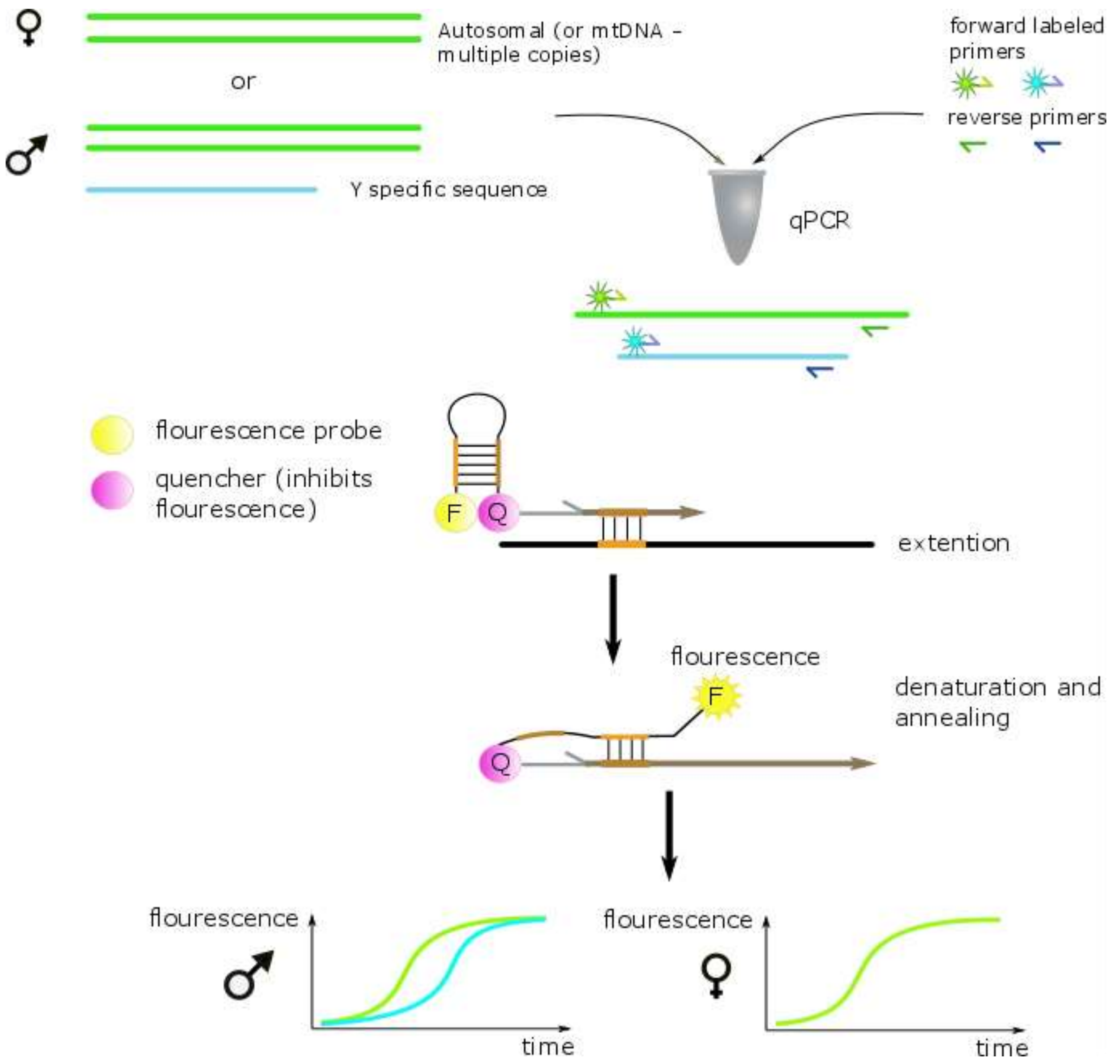

qPCR –quantitative polymerase chain reaction; IPC – internal positive control; mtDNA – mitochondrial DNA

qPCR amlification of Y specific and IPC sequence with flourescent probe

Method	TaqMan qPCR
Sex sequence variant	Y specific sequence + IPC
Detection	qPCR – flourescence of digested probe
References	O'Neill et al., 2013

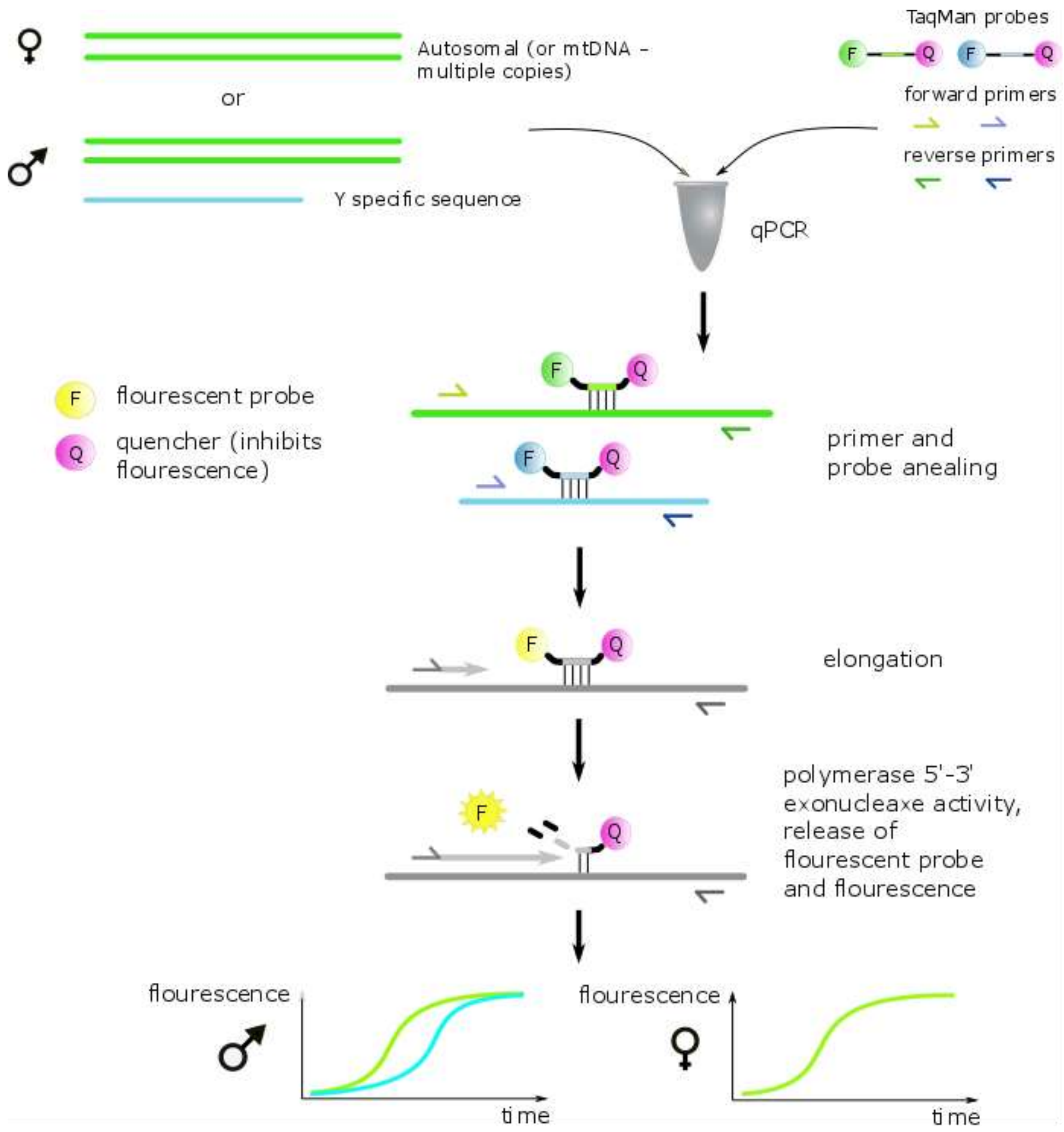

qPCR –quantitative polymerase chain reaction; IPC – internal positive control; mtDNA – mitochondrial DNA

LAMP reaction of Y (male) specific and IPC region

Method	LAMP reaction of Y specific and IPC region
Sex sequence variant	Y specific sequence + IPC
Detection	Visualisation of white precipitate
References	Hirayama et al., 2004; Hirayama et al., 2006

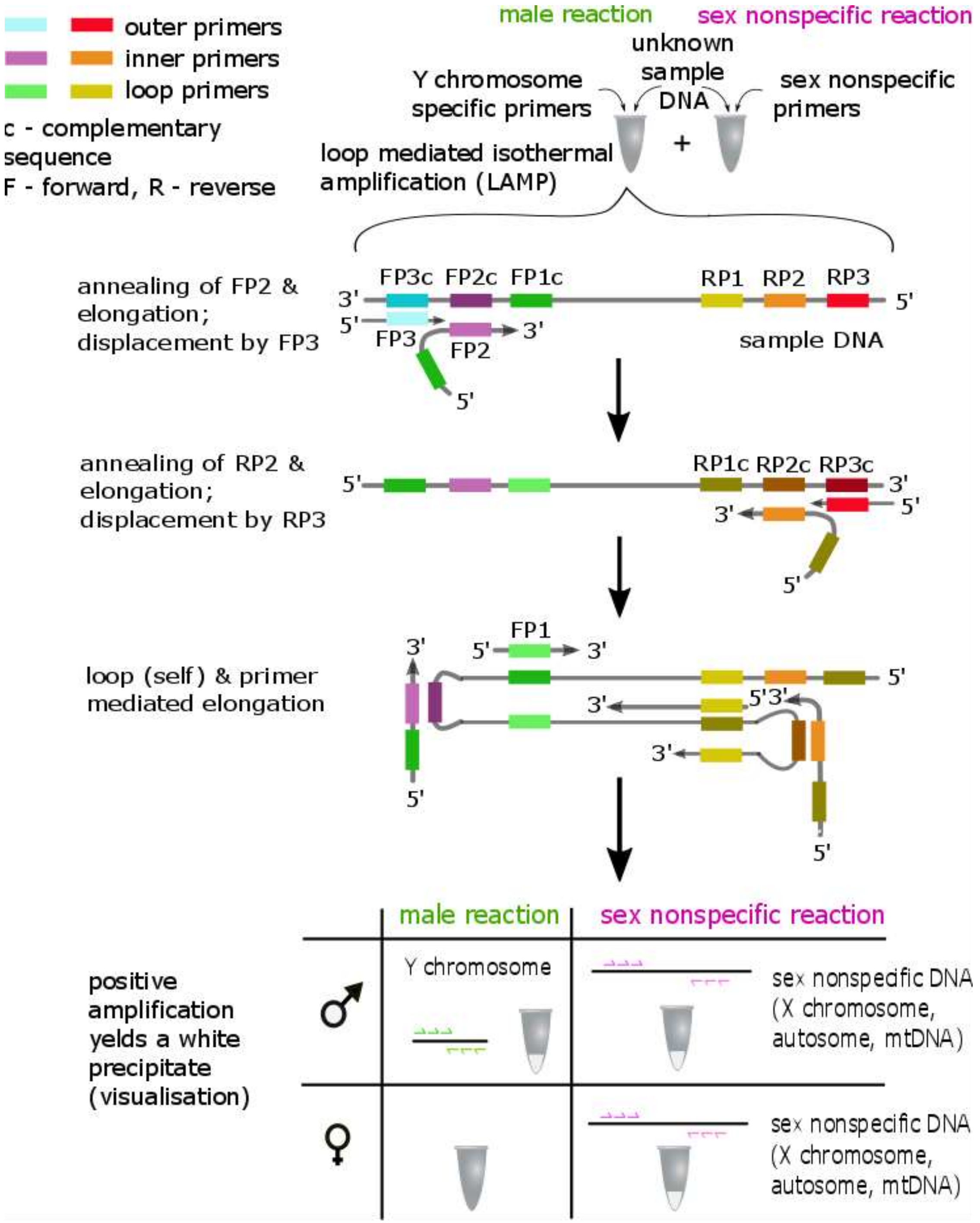

IPC – internal positive control; FP – forward primer; RP – reverse primer; LAMP – loop mediated isothermal amplification; mtDNA – mitochondrial DNA

Electrophoreses

- Gel (agarose, polyacrylamide (PAGE), piperazine diacrylamide,…) – detection with coloring of amplicons after amplification
- Capillary – detection with tagging primers and thus amplicons during amplification

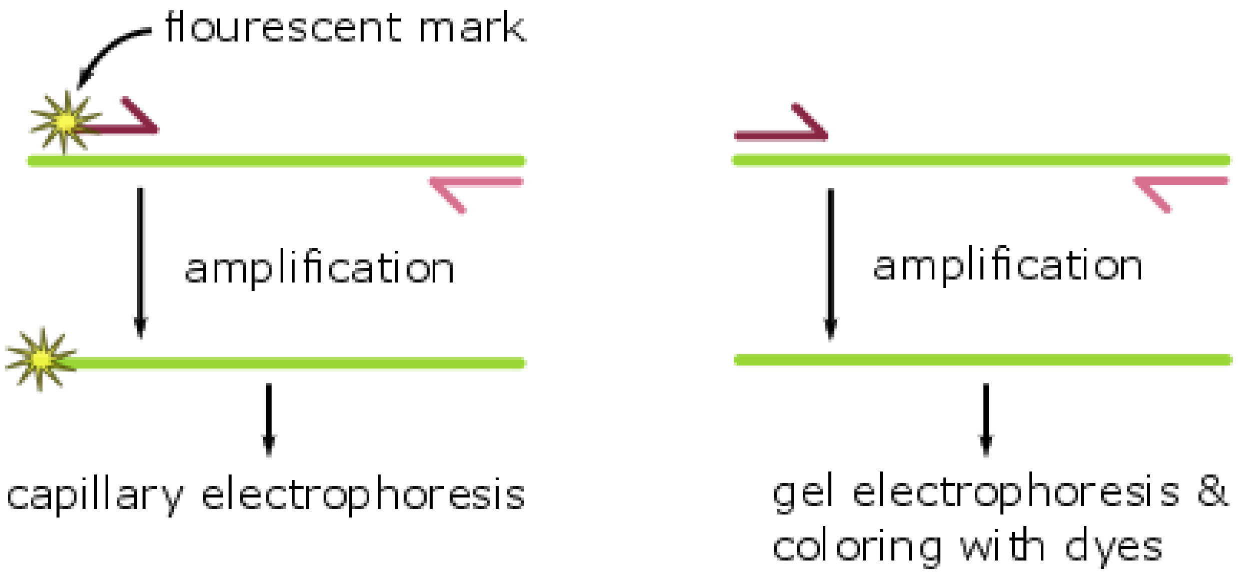

Sex specific sequence variants

- Length
- Sequence
- Other (dose variation, GC vs. AT content)

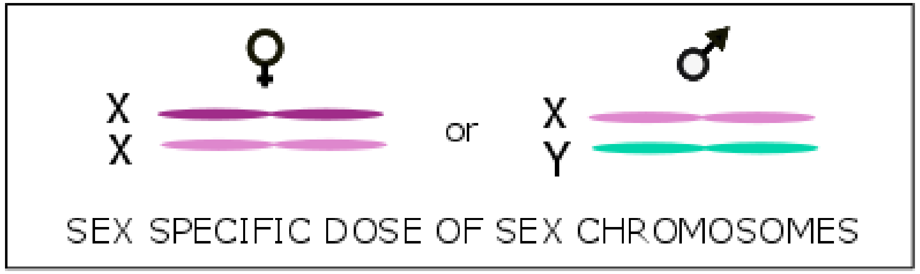

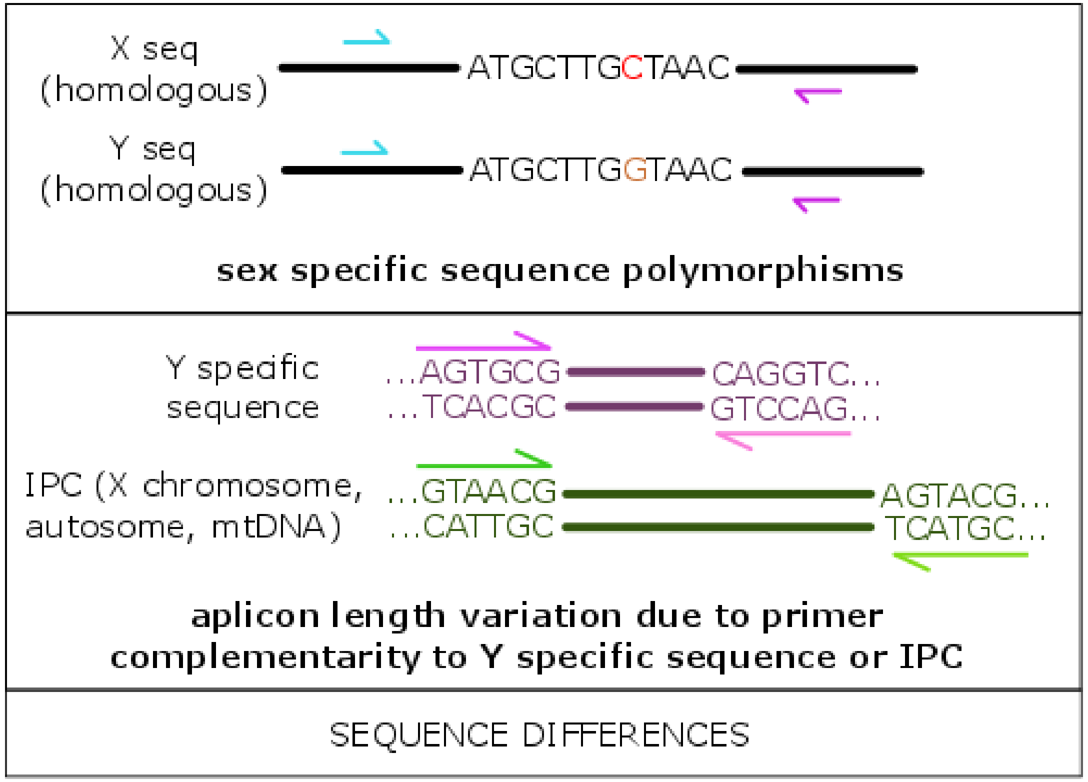

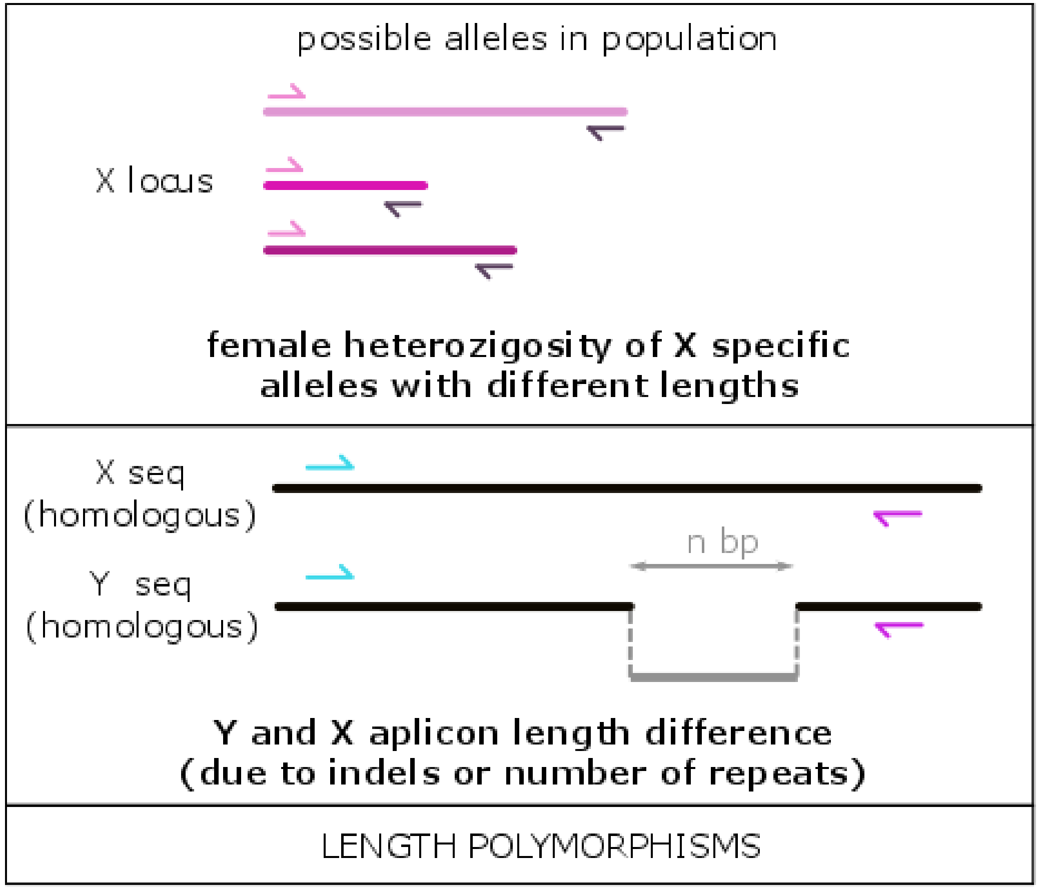

seq – sequence;
IPC – internal
positive control

- Length polymorphisms

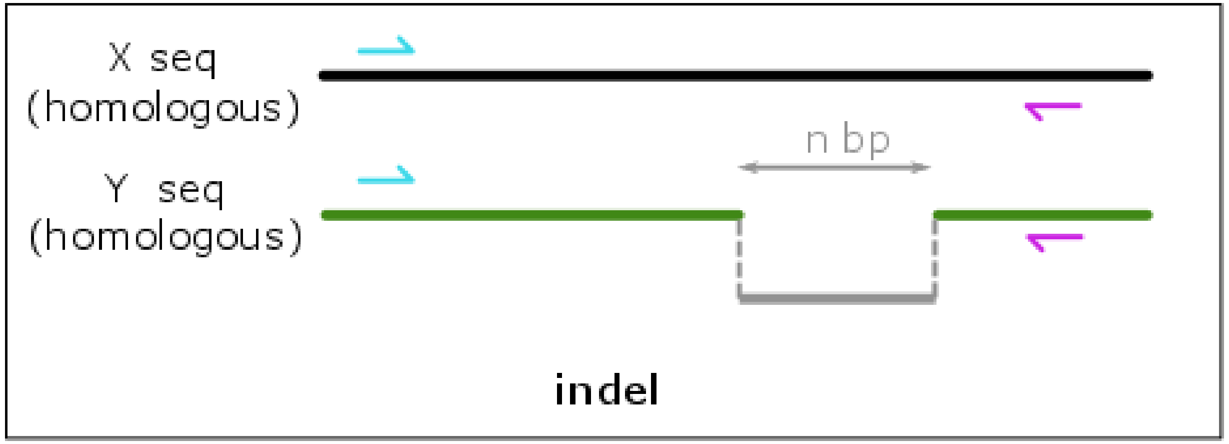

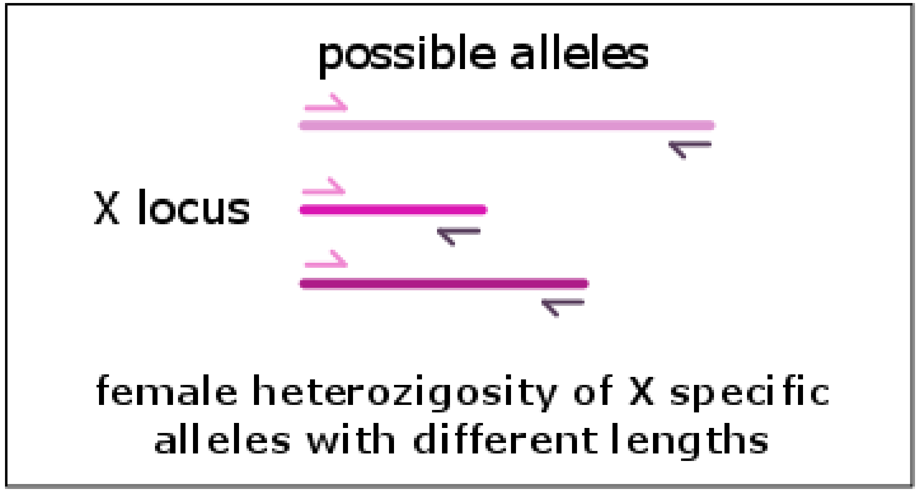

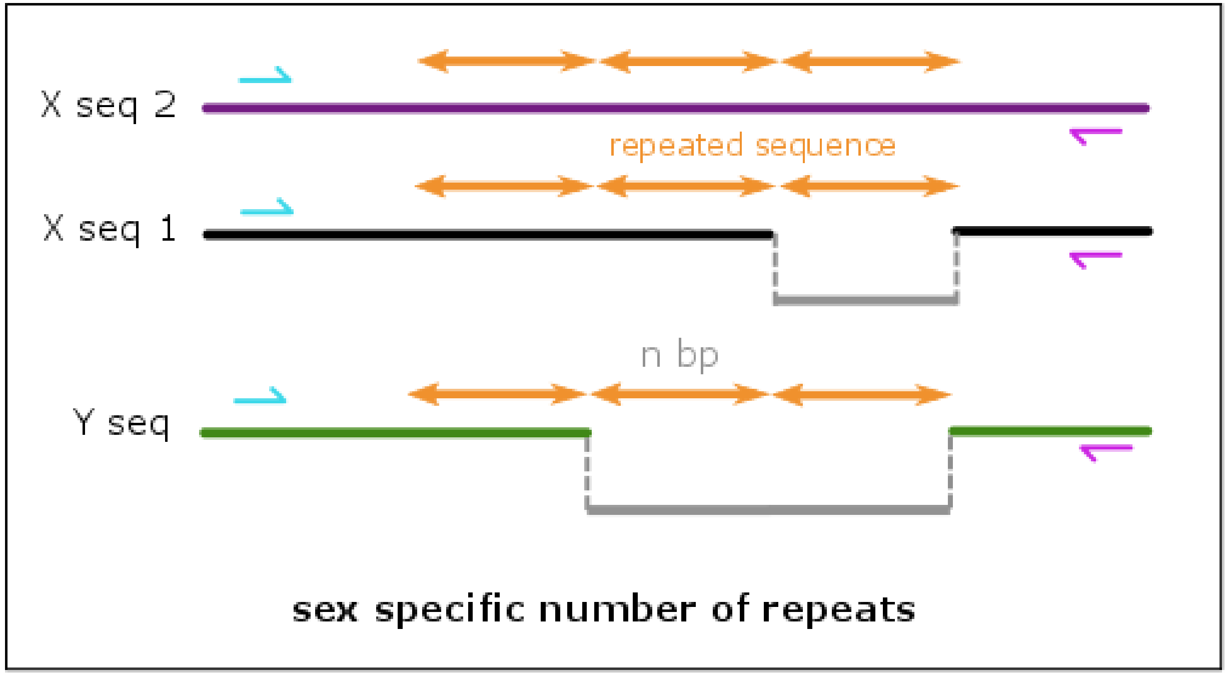

seq – sequence

Separation of different length fragments on the gel due to an indel in X and Y specific gene

Method	PCR of sequences of different length due to indels
Sex sequence variant	Indel in homologous X and Y chromosome sequences
Detection	Gel electrophoresis
References	Akane et al., 1991; Sullivan et al., 1993; Haas-Rochholz, 1997; Rao and Totey, 1999; Williams et al., 2004; Clapcote and Roder, 2005; Statham et al., 2007; Mchale et al., 2008; Tschentscher, 2008; Pages et al., 2009; Sekiquchi et al., 2010; Morikawa et al., 2011; Murata et al., 2011; Jacot et al., 2013; McFarlane et al., 2013; Tozzo et al., 2013; Liu et al., 2015; Pratner et al., 2016; Tavares et al., 2016

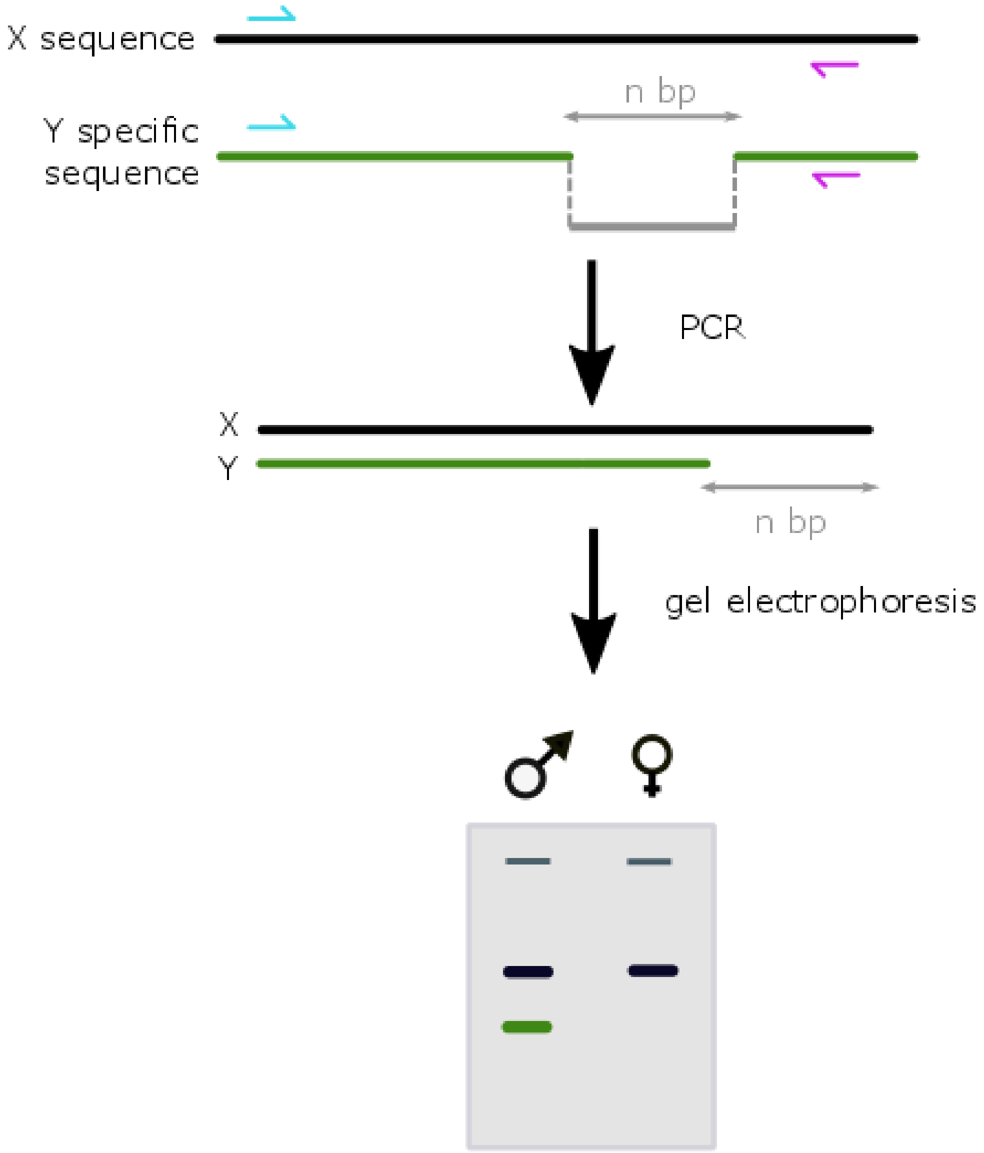

PCR – polymerase chain reaction

Detection of PCR products with capillary electrophoresis

Method	Detection of different length amplicons with capillary electrophoresis
Sex sequence variant	Indel in homologous X and Y chromosome sequences or Y specific sequence+IPC
Detection	capillary electrophoresis
References	Steinlechner et al., 2002; Mchale et al., 2008; Kastelic et al., 2009; Codina et al., 2009; Sekiquchi et al., 2010; Jacot et al., 2013

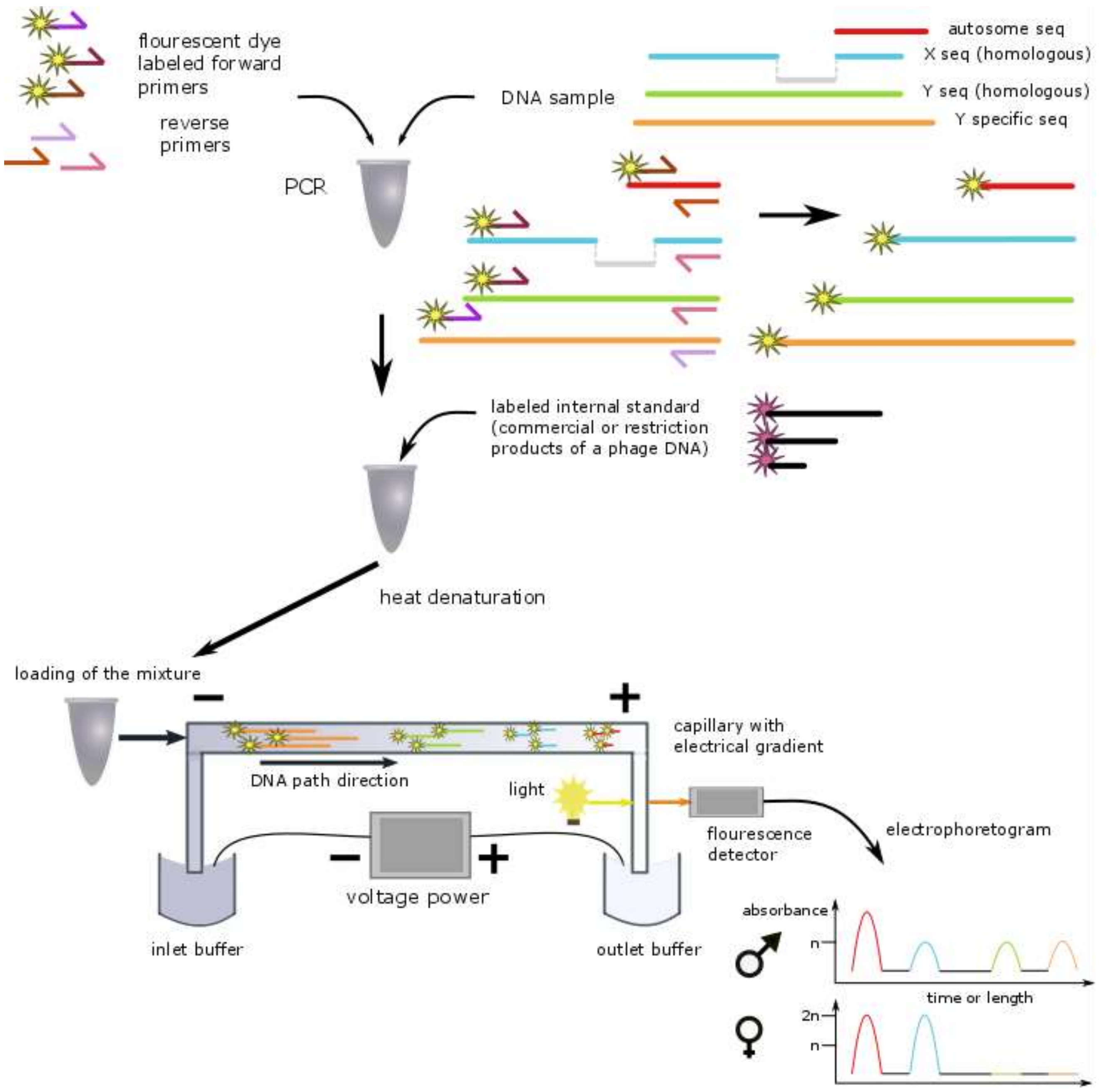

PCR – polymerase chain reaction; seq - sequence

Separation of different length fragments on the gel due to specific number of repeats in X and Y specific regions

Method	PCR of sequences with sex specific number of repeats
Sex sequence variant	X and Y specific number of repeats
Detection	Gel (agarose/acrylamide) electrophoresis
References	Chen et al., 1994

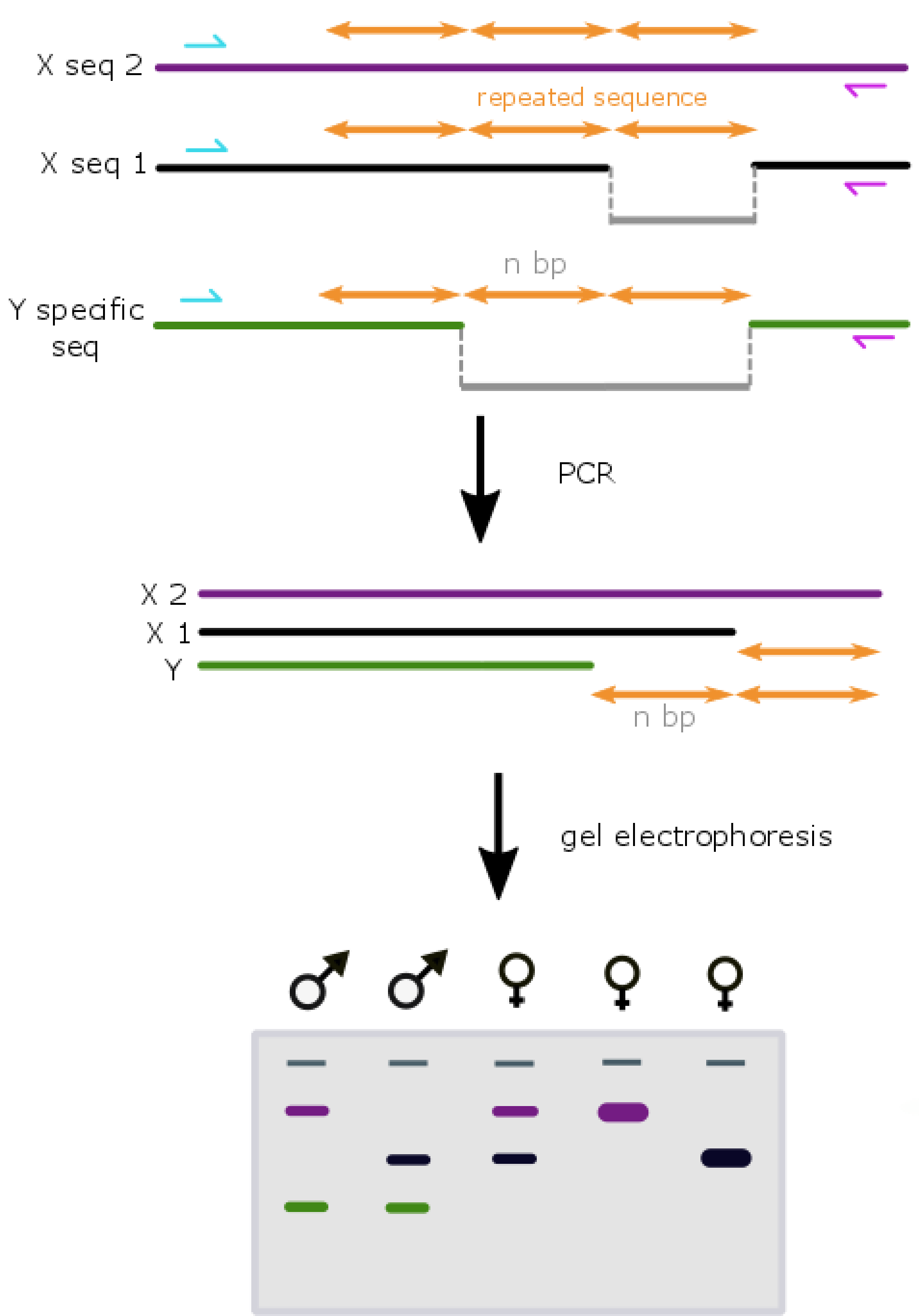

PCR – polymerase chain reaction; seq – sequence

Female heterozigosity of X chromosome loci

Method	PCR of differently long X chromosome alleles
Sex sequence variant	Female heterozigosity of X loci
Detection	Gel or capillary electrophoresis
References	Codina et al., 2009

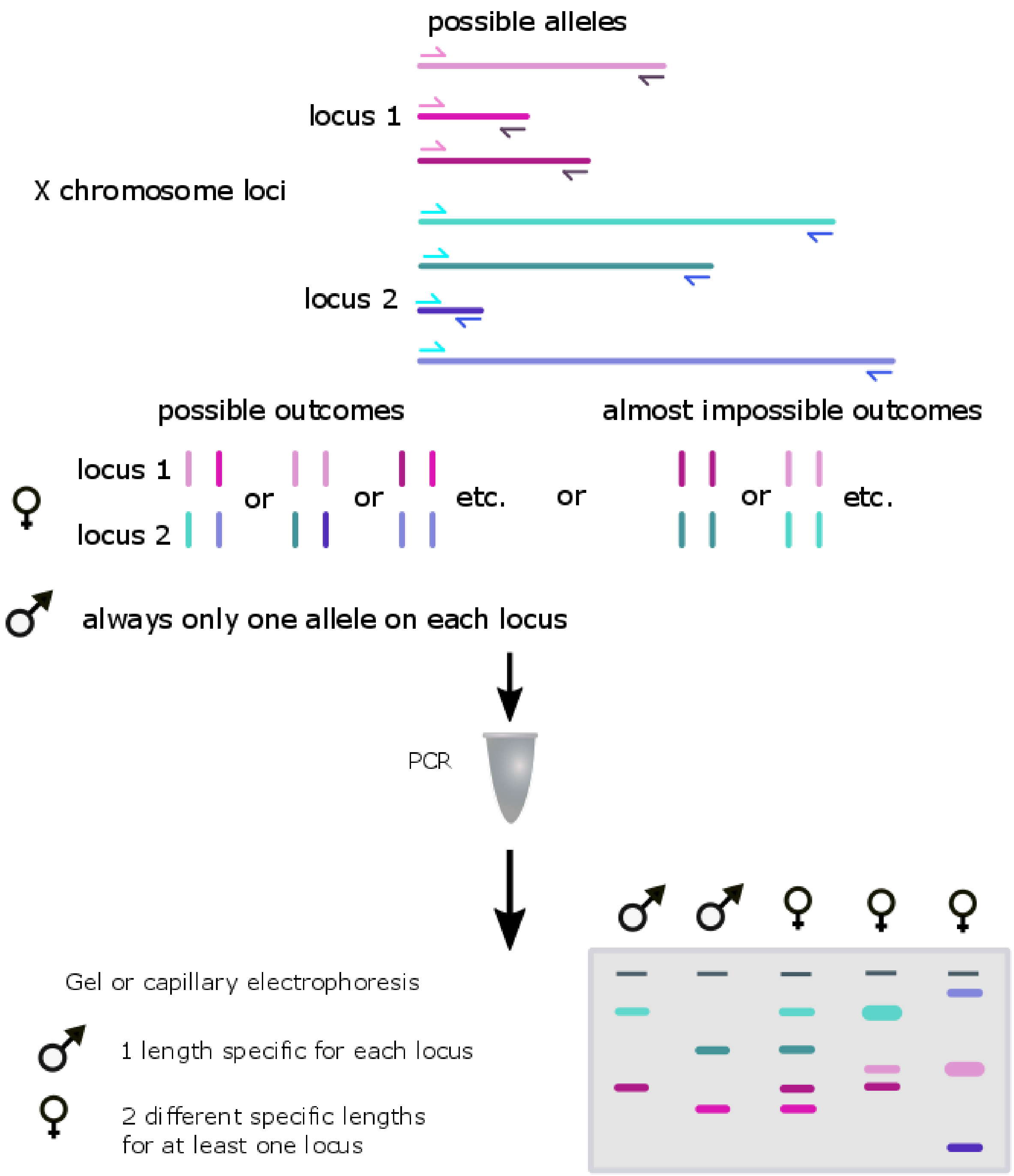

PCR – polymerase chain reaction

Sequencing & double peaks (due to indel and thus sequencing frame shift) identification

Method	PCR + Sanger sequencing
Sex sequence variant	Indel in homologous X and Y chromosome sequences
Detection	Sanger sequencing

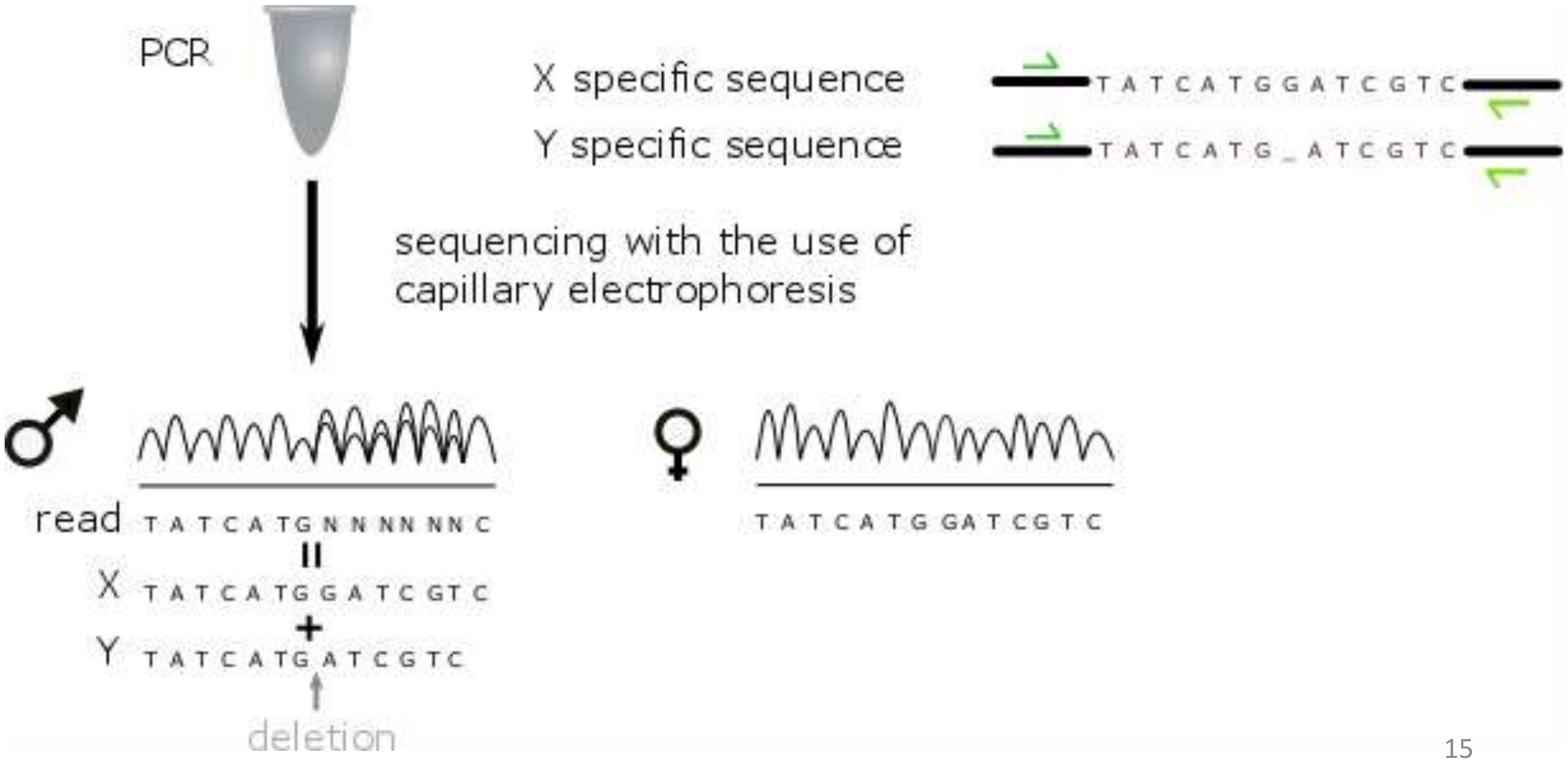

PCR – polymerase chain reaction; SSSP – sex specific sequence polymorphism

- Sequence differences

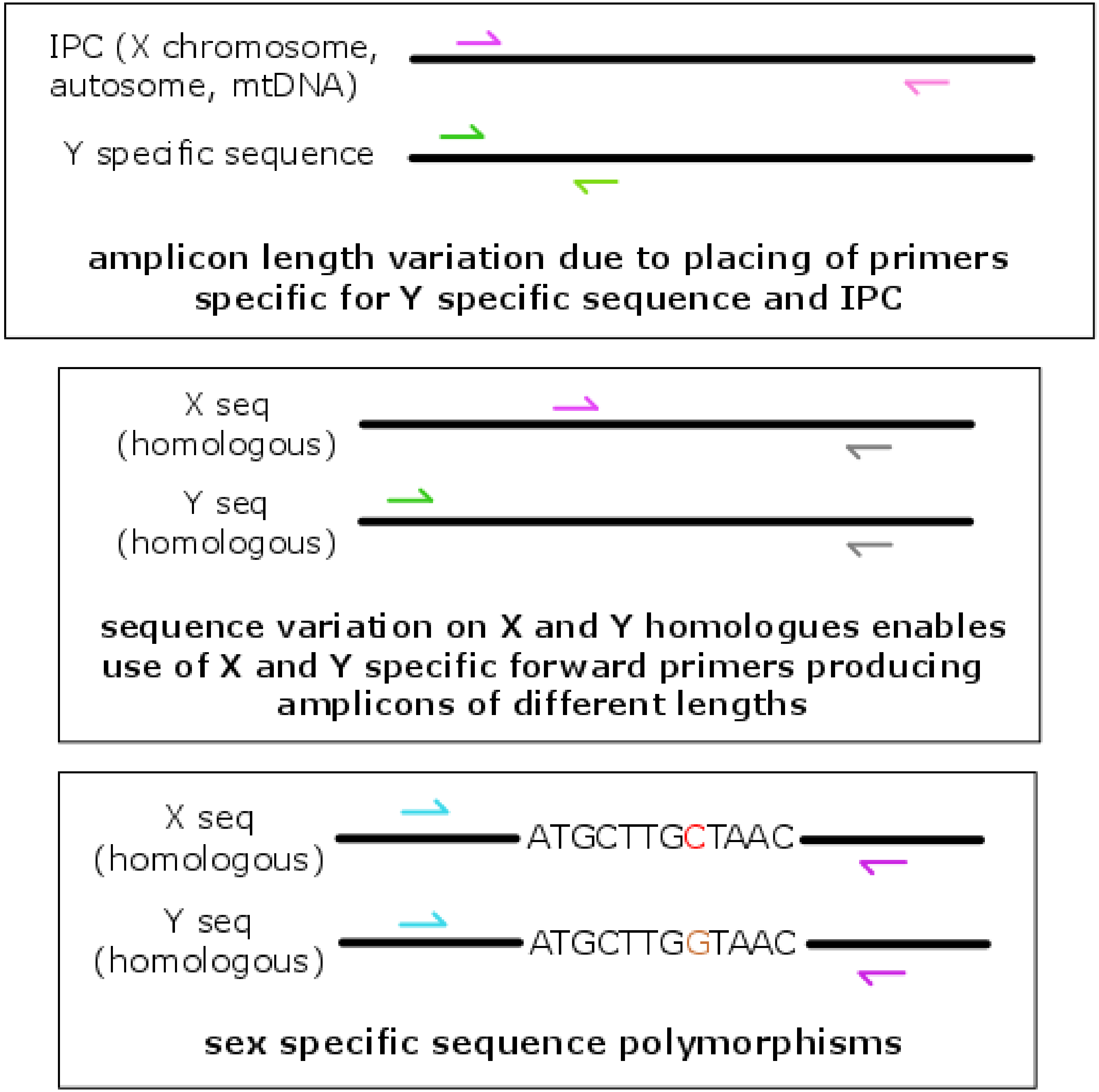

seq – sequence; mtDNA – mitochondrial DNA;
IPC – internal positive ontrol

Amplification of Y specific and internal positive control (IPC) followed by gel electrophoresis; length variation due to placing of sequence specific primers

Method	PCR of Y specific and IPC sequences with different lengths
Sex sequence variant	Y specific sequence + IPC
Detection	Gel electrophoresis
References	Akane et al., 1991; Yano, 1993; Rao and Totey, 1999; Weikard et al., 2001; Mara et al., 2004; Fu et al., 2007; Statham et al., 2007; Mchale et al., 2008; Pages et al., 2009; Katsushima et al., 2010; Sekiquchi et al., 2010; Luptakovaet al., 2011; Morikawa et al., 2011; Gokulakrishna et al., 2012; Gorrell et al., 2012; Korstian et al., 2013; Tozzo et al., 2013; Wedrowicz et al., 2013; Prugnard 2016; Salabi et al., 2014; Pratner et al., 2016

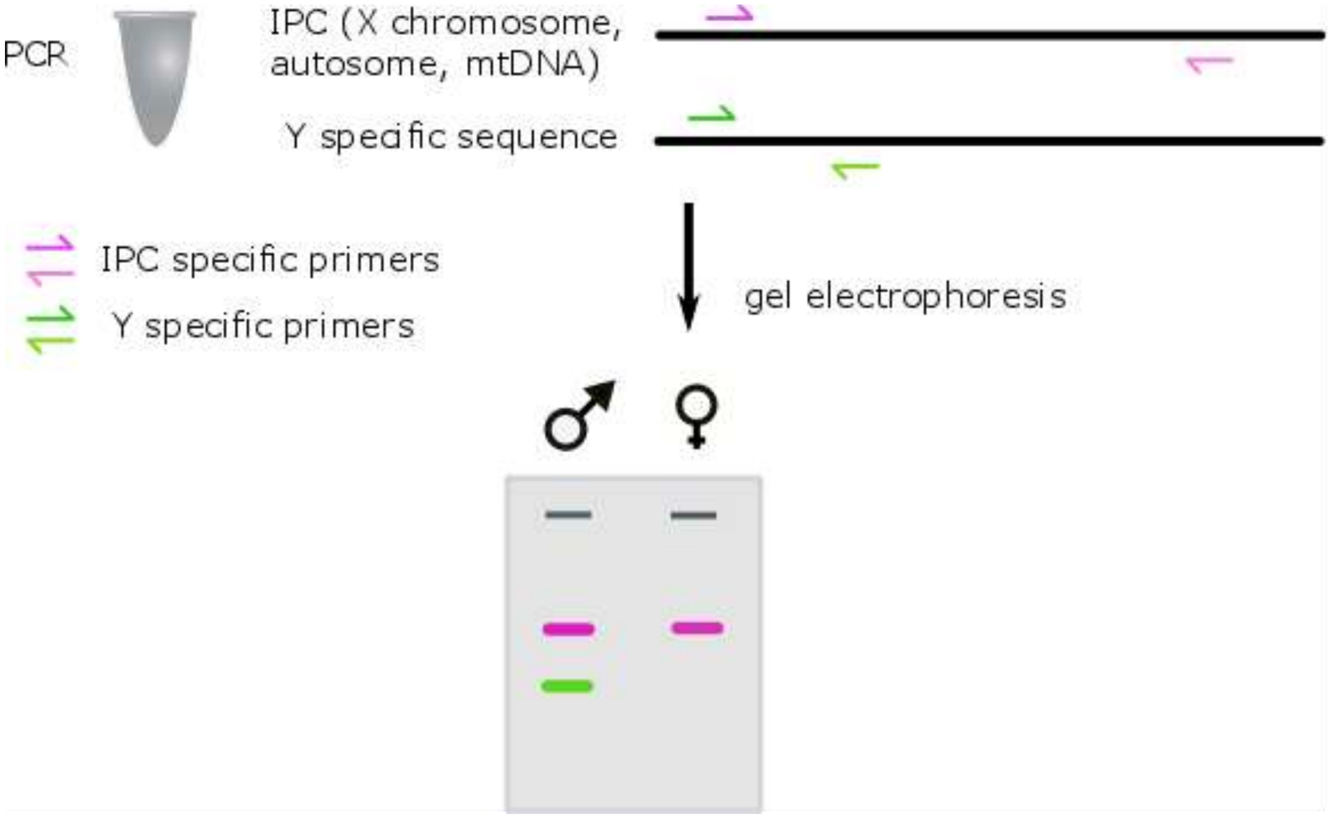

PCR – polymerase chain reaction; IPC – internal positive control; mtDNA – mitochondrial DNA; IPC – internal positive control

Amplification of two Y specific and one internal positive control (IPC) followed by gel electrophoresis; length variation due to placing of sequence specific primers

Method	Double male positive PCR of Y specific and IPC sequences with different lengths
Sex sequence variant	Two Y specific sequences + IPC
Detection	Gel electrophoresis
References	Ahlering et al., 2011; Bidon et al., 2013; Jacot 2013; Campos et al., 2014

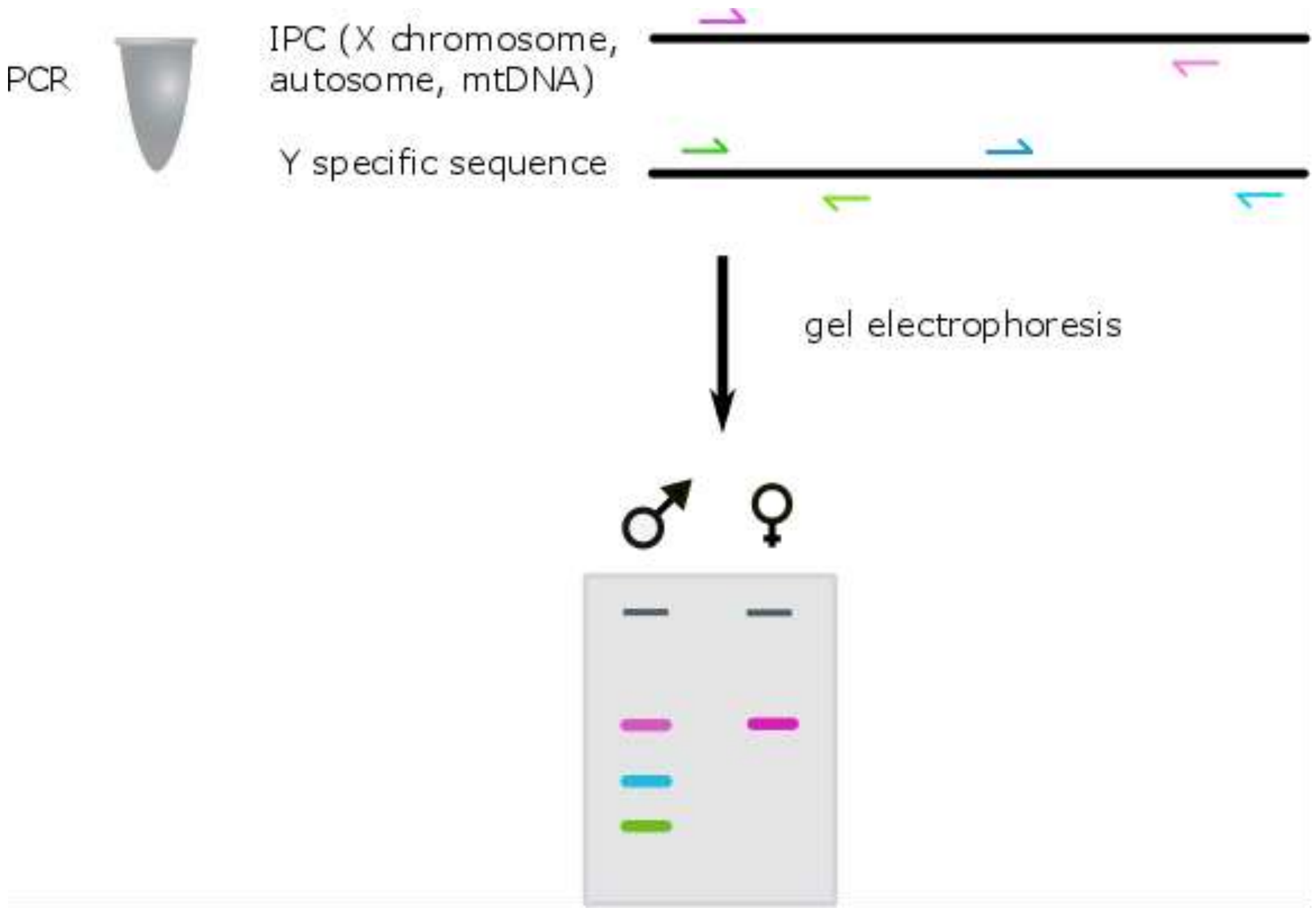

PCR – polymerase chain reaction; IPC – internal positive control; mtDNA – mitochondrial DNA

Sex chromosome alelle specific amplification; length variation due to placing of sequence specific primers - ARMS

Method	PCR of homologous X and Y specific sequences with one common and two sequence specific primers
Sex sequence variant	Different length of X and Y amplicons due to primer annenaling
Detection	Gel electrophoresis
References	Li et al., 2011; Tsai et al., 2011

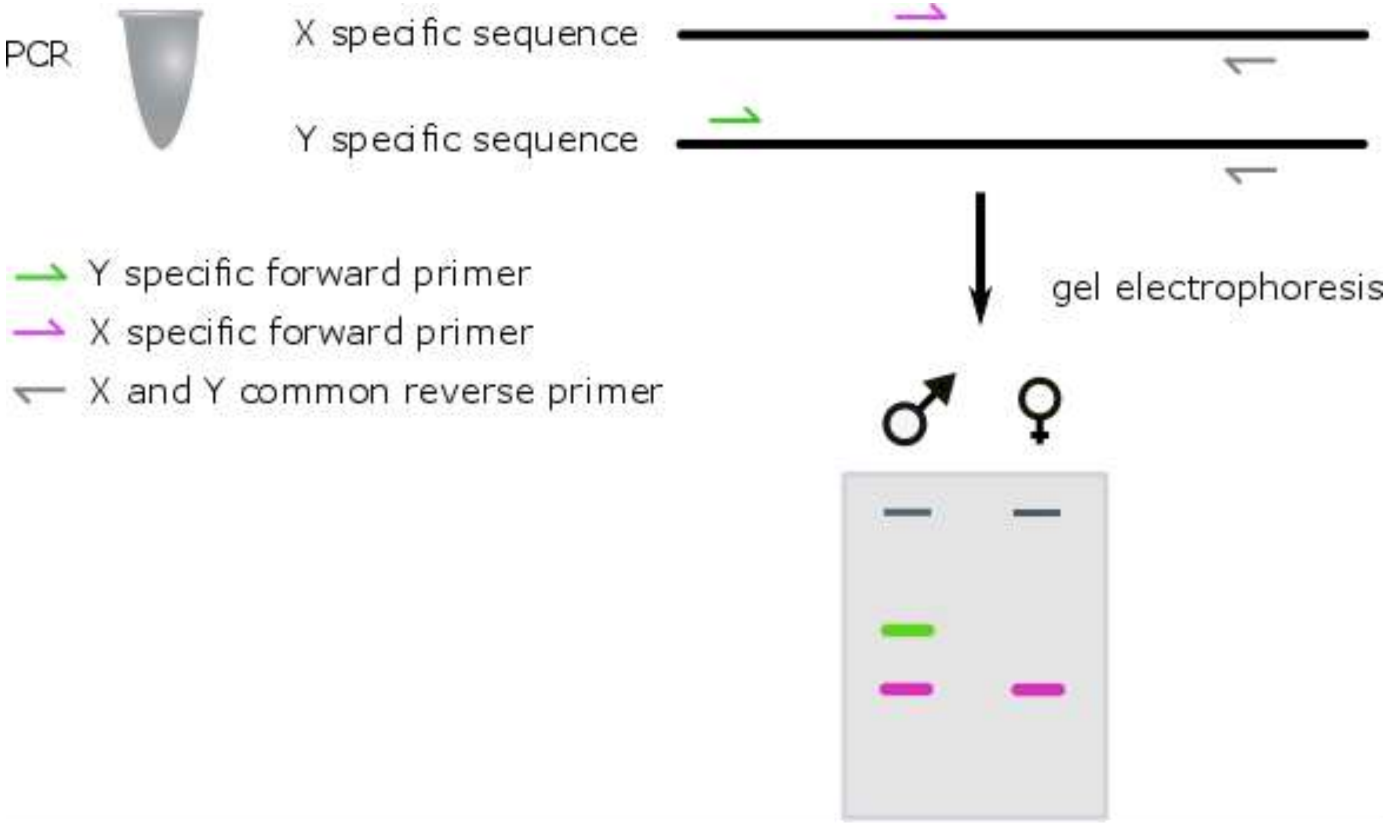

PCR – polymerase chain reaction; ARMS – amplification refractory mutation system

PCR-RFLP analysis of sex specific sequence polymorphism

Method	PCR-RFLP
Sex sequence variant	Sex specific sequence polymorphism
Detection	Gel electrophoresis
References	Gutiérrez-Adán et al., 1997; Pande and Totey1998; Saravanan et al., 2003; Ortega et al., 2004; Statham et al., 2007; Sekiquchi et al., 2010; Fontanesi et al. , 2011; Martinelli et al., 2011

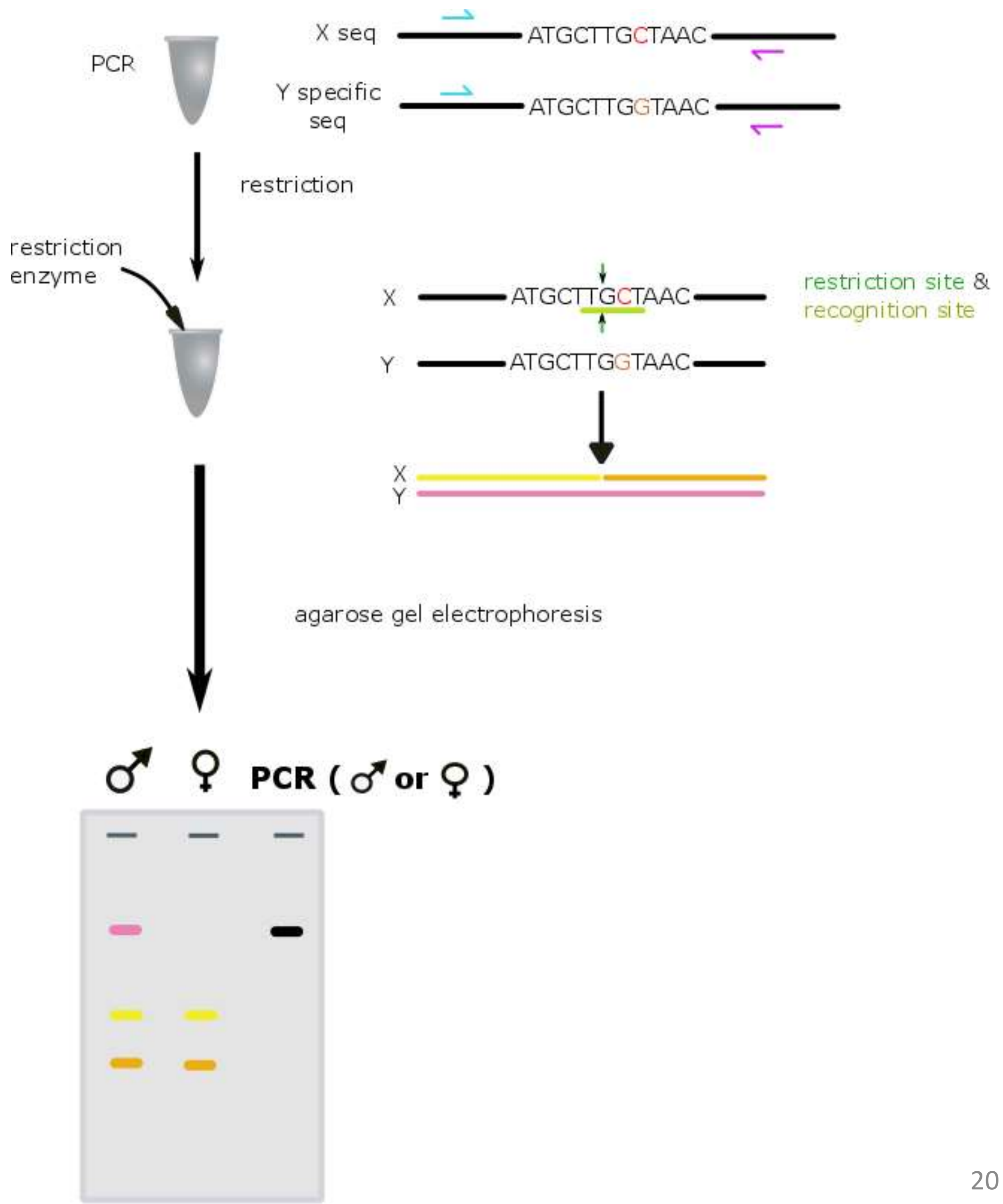

PCR – polymerase chain reaction: RFLP - restriction fragment length polymorphism; seq – sequence

Sequencing & double peaks (due to SSSP) identification

Method	PCR + Sanger sequencing
Sex sequence variant	SSSP
Detection	Sanger sequencing
References	Statham et al., 2007; Gibbon et al., 2009

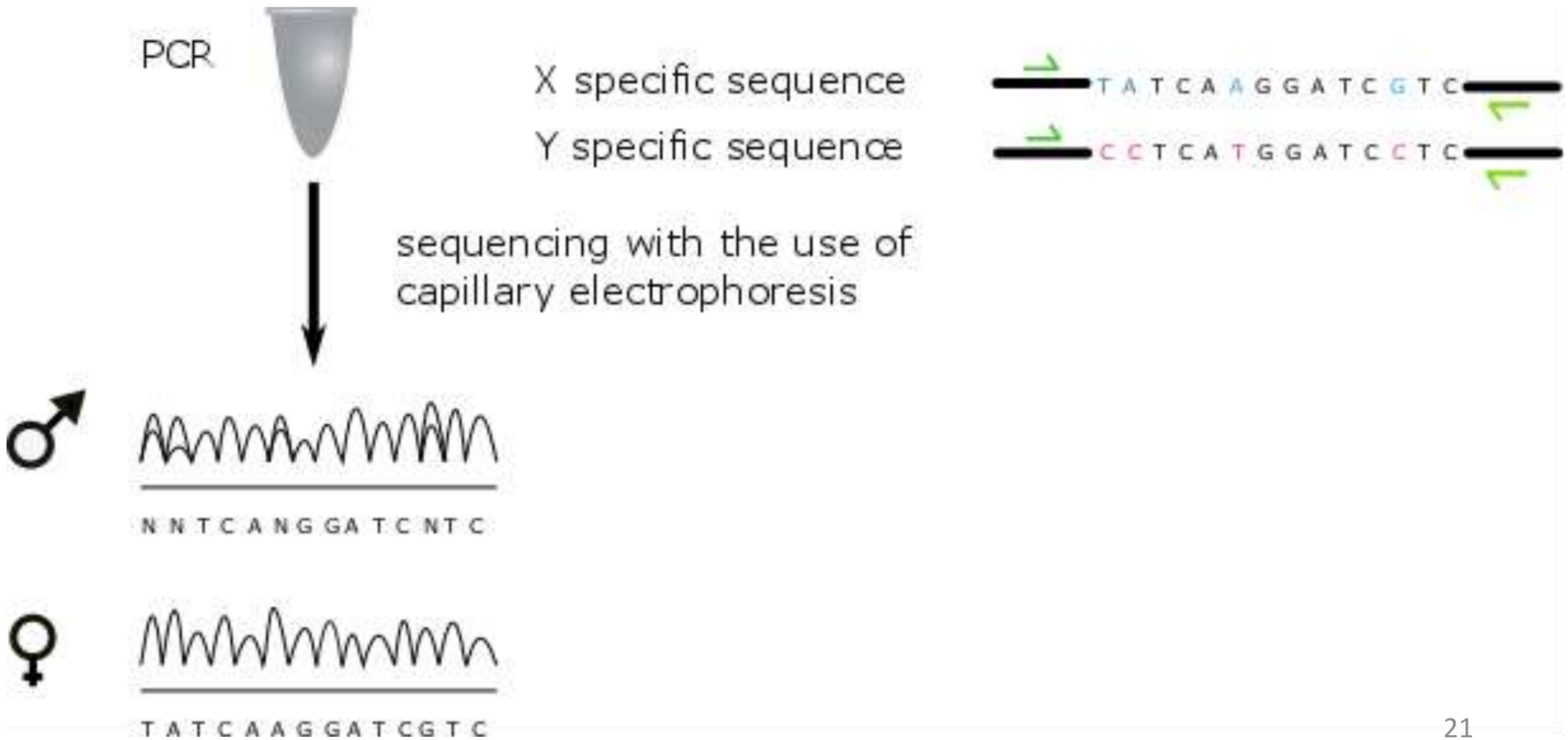

PCR – polymerase chain reaction;
SSSP – sex specific sequence polymorphism

Pyrosequencing & detection of higher/lower/absent peaks due to SSSPs and indels in X and Y homologous sequences

Method	PCR + pyrosequencing
Sex sequence variant	SSSP or indel
Detection	pyrosequencing
References	Tschentscher, 2008; Li et al., 2012

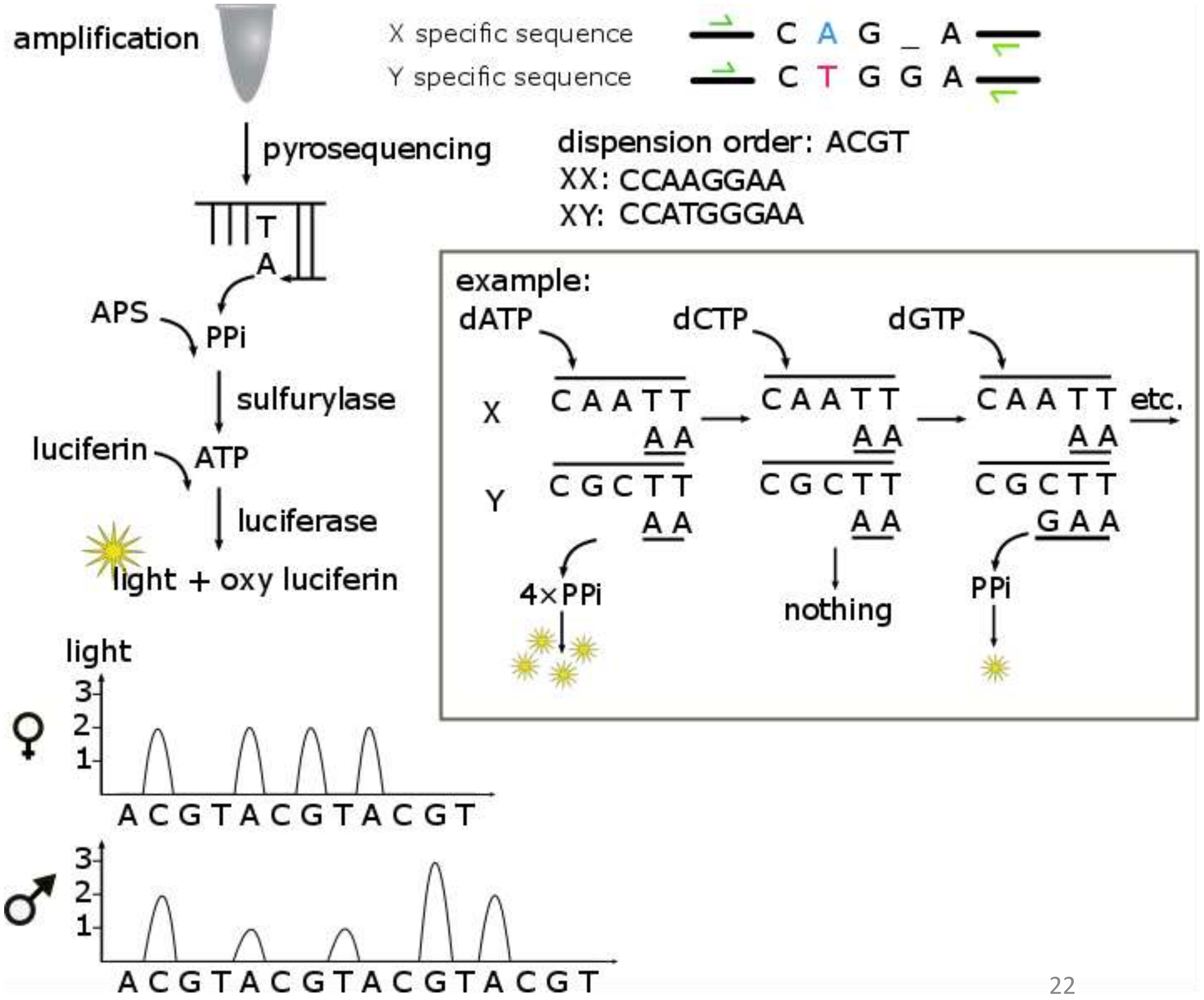

PCR – polymerase chain reaction; APS – adenosine 5'-phosphosulfate; PPi – pyrophosphate; SSSP – sex specific sequence polymorphism

• Other polymorphisms

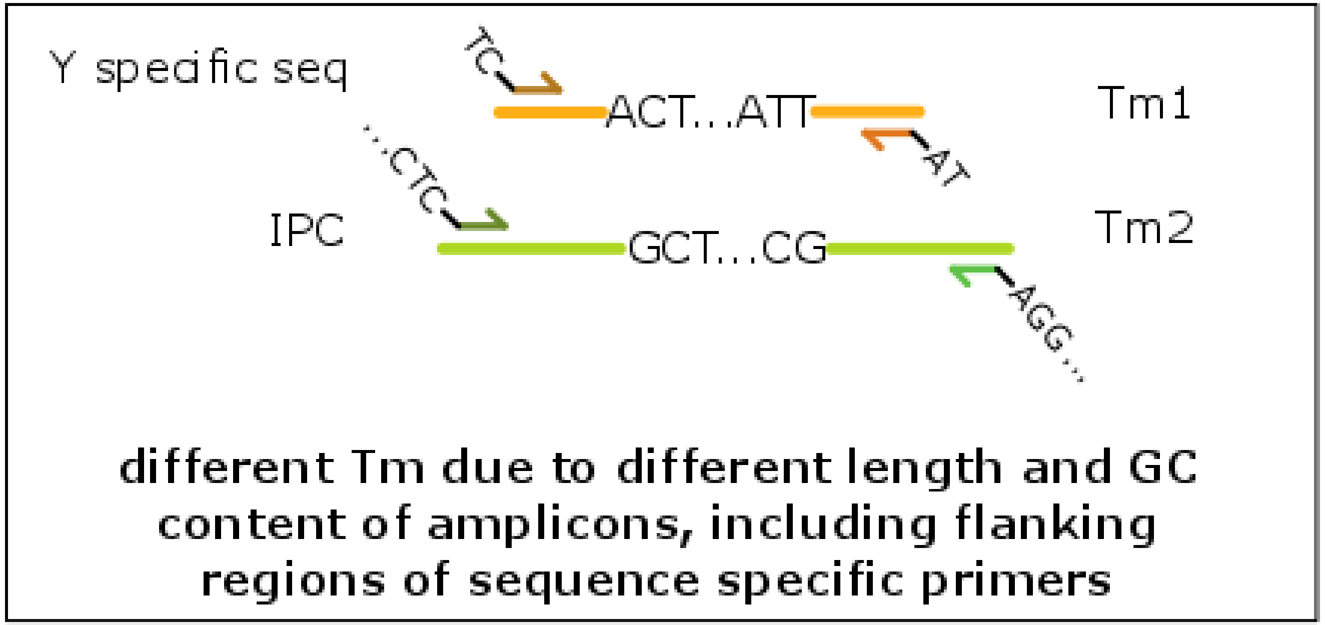

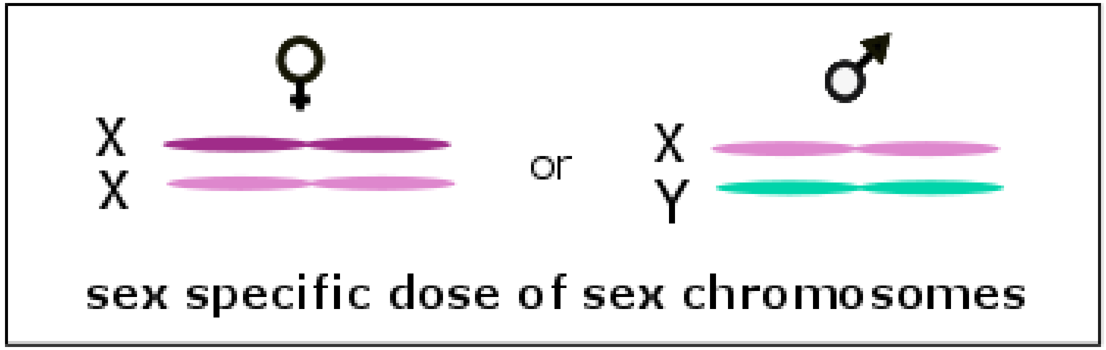

IPC – internal positive control; seq – sequence; Tm – melting temperature

Shotgun sequencing and calculation of Ry and Rx from number of alignments to sex chromosomes due to sex specific dose variation

Method	Shotgun sequencing + Ry and Rx
Sex sequence variant	Sex specific dose variation
Detection	Counting of chromosome specific sequences and calculation
References	Mittnik et al., 2016

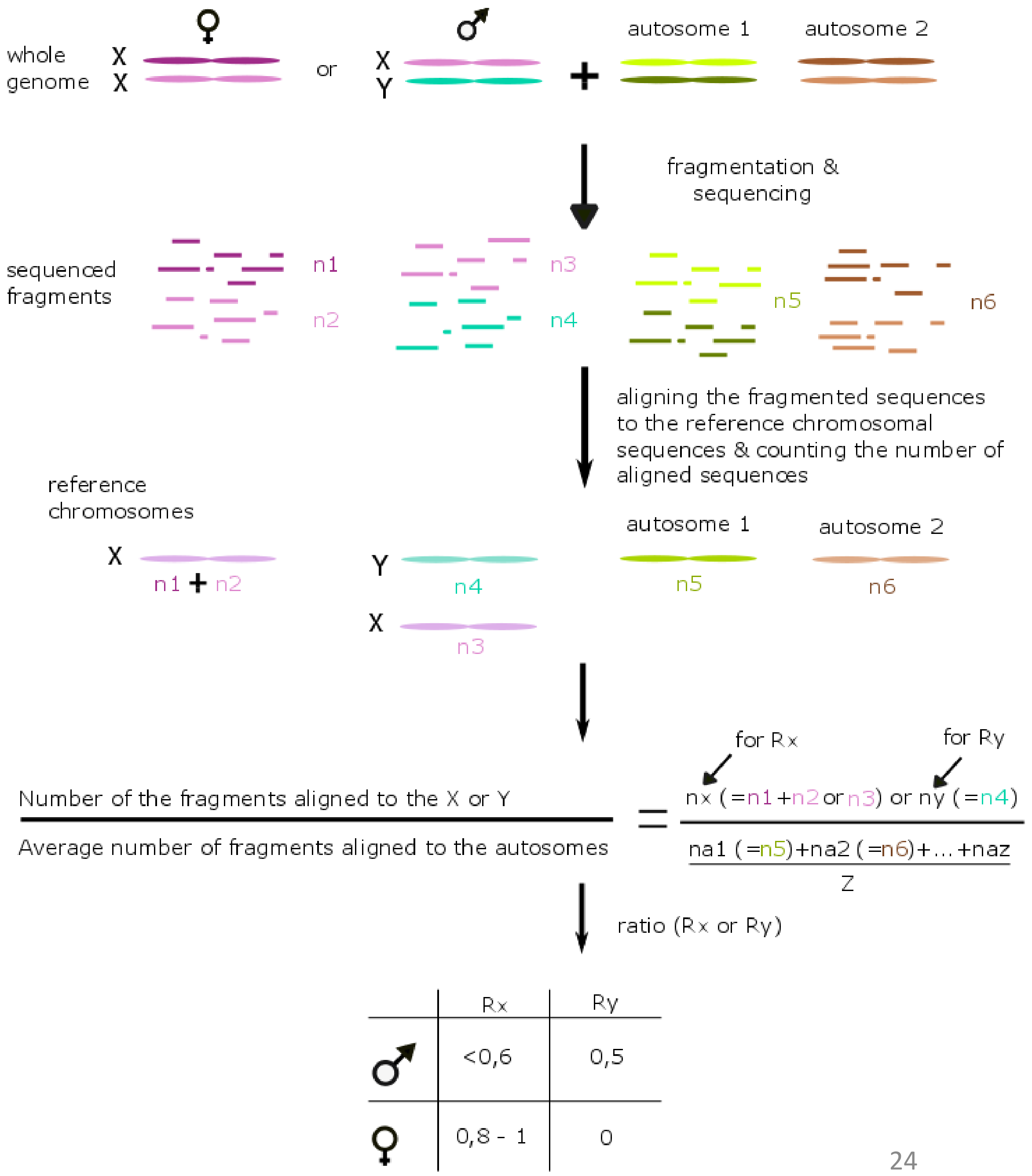

R – the ratio of alignment to a specific chromosome and an autosome; PCR – polymerase chain reaction

Analysis of a sex specific melting curve; Y specific sequence and IPC

Method	PCR-HRM
Sex sequence variant	Y specific sequence and IPC vary in melting temperature
Detection	electrophoresis
References	Madel et al., 2016

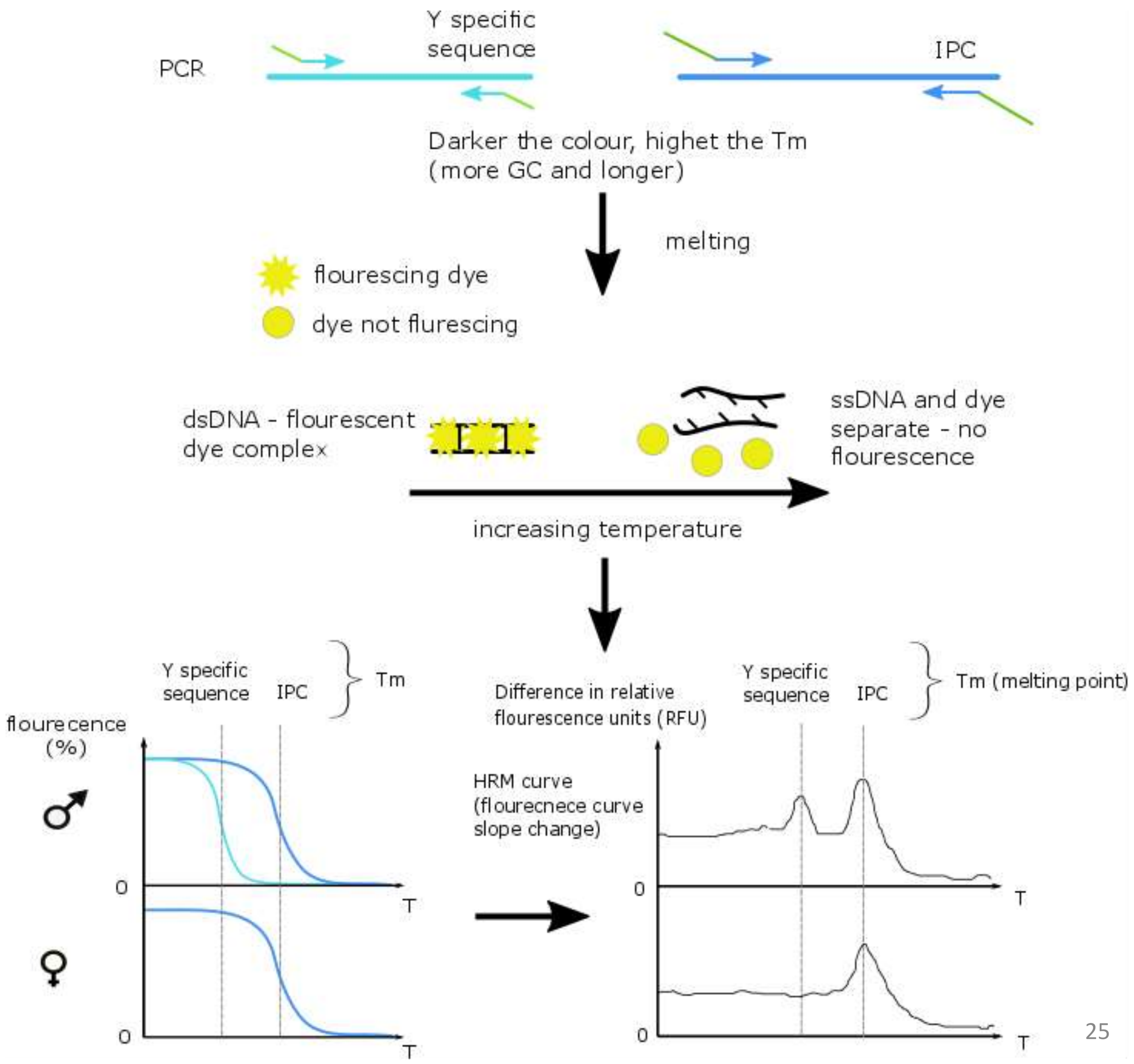

dsDNA – double stranded DNA; HRM – high resolution melting; IPC – internal positive control; PCR – polymerase chain reaction; Tm – melting temperature; ssDNA – single stranded DNA
