# Supplementary material for: Genetic sex determination assays in 53 mammalian species: Literature analysis and guidelines for reporting standardization
Source: Ecol Evol. 2017 Dec 13;8(2):1009–18. doi: 10.1002/ece3.3707 (PMC5773321; doi:10.1002/ece3.3707)
Supplement: Supplementary file 2 [file ECE3-8-1009-s002.docx]

Table S1. Summary of sexing assays, described in 58 publications. Each test includes the following information: animal species, gene / sequence, method used for sex determination, sex specific sequence variants (SSSVs), author, year of publication and its ID. Each line represents one assay used on one animal species. In case when one assay was developped for multiple species, each of them is presented seperately.

| Species, common name | Species, scientific name, strains in mouse | Species (NCBI) id | Gene/ sequence | Gene ID (NCBI) (if available) | SSSV | Method | Author | Year | Publication ID |
| --- | --- | --- | --- | --- | --- | --- | --- | --- | --- |
| Koala | *Phascolarctos cinereus* | 38626 | *SRY,*  *G6PD* | NA,  110200335 | Y chromosome specific fragment | multiplex PCR of male specific and control region | Wedrowicz et al. | 2013 | PMID: 23582171 |
| Forest elephant | *Loxodonta cyclotis* | 99490 | *SRY,*  *AMELY,*  *PLP* | NA,  AY823322,  AY823382–AY823385 | Y chromosome specific fragment | double male specific positive PCR test | Ahlering et al. | 2011 | PMID: 21635697 |
| Savannah elephant | *Loxodonta africana* | 9785 | *SRY,*  *AMELY,*  *PLP* | NA,  AY914339,  PLP: AB362890 and AY823279–AY823381 | Y chromosome specific fragment | double male specific positive PCR test | Ahlering et al. | 2011 | PMID: 21635697 |
| Asian elephant | *Elephas maximus* | 9783 | *SRY,*  *AMELY,*  *PLP* | AF180946,  AY823325,  AY823386–AY823387 | Y chromosome specific fragment | double male specific positive PCR test | Ahlering et al. | 2011 | PMID: 21635697 |
| Dugong | *Dugong dugon* | 29137 | *SRY,*  *ZFY*  *ZFX* | EU078404,  EU078400,  EU078399 | Y chromosome specific fragment, indel | multiplex PCR of male specific and control region | Mchale et al. | 2008 | PMID: 21585866 |
| West indian manatee | *Trichechus manatus* | 9778 | *SRY,*  *ZFY,*  *ZFX* | AF180946,  EU078402,  EU078401 | Y chromosome specific fragment, indel | multiplex PCR of male specific and control region | Mchale et al. | 2008 | PMID: 21585866 |
| Maned three-toed sloth | *Bradypus torquatus* | 227087 | *ZFY,*  *ZFX* | GU187041,  GU187042 | SSSP | PCR-RFLP | Martinelli et al. | 2010 | PMID: 21565080 |
| Brown-throated sloth | *Bradypus variegatus* | 9355 | *ZFY,*  *ZFX* | GU187044,  GU187043 | SSSP | PCR-RFLP | Martinelli et al. | 2011 | PMID: 21565080 |
| European rabbit | *Oryctolagus cuniculus* | 9986 | *ZFY,*  *ZFX* | AM778418,  100350200 | SSSP | PCR-RFLP | Fontanesi et al. | 2008 | PMID: 21586025 |
| Mountain hare | *Lepus timidus* | 62621 | *ZFY,*  *ZFX* | AM778432,  AM778431 | SSSP | PCR-RFLP | Fontanesi et al. | 2008 | PMID: 21586025 |
| European brown hare | *Lepus europaeus* | 9983 | *ZFY,*  *ZFX* | AM778430,  AM778429 | SSSP | PCR-RFLP | Fontanesi et al. | 2008 | PMID: 21586025 |
| American beaver | *Castor canadensis* | 51338 | ZFX,  *ZFX* | NA | indel | PCR identification of sex specific length variation due to indels | Williams et al. | 2004 | WOS:000225950800015 |
| Naked mole rat | *Heterocephalus glaber* | 10181 | *Ddx3y (DBY),*  16S rDNA | AB474907,  NA | Y chromosome specific fragment | duplex PCR of male specific and control region | Katsushima et al. | 2010 | PMID: 21565015 |
| Eastern grey squirrel | *Sciurus carolinensis* | 30640 | *Smcy,*  microsatellite | JQ361902,  NA | Y chromosome specific fragment | duplex PCR of male specific and control region | Gorrell et al. | 2012 | PMID: 22726203 |
| North American red squirrel | *Tamiasciurus hudsonicus* | 10009 | *Smcy,*  microsatellite | JQ361901,  NA | Y chromosome specific fragment | duplex PCR of male specific and control region | Gorrell et al. | 2012 | PMID: 22726203 |
| Columbian ground squirrel | *Urocitellus columbianus* | 50862 | *Smcy,*  microsatellite | JQ361904,  NA | Y chromosome specific fragment | duplex PCR of male specific and control region | Gorrell et al. | 2012 | PMID: 22726203 |
| Arctic ground squirrel | *Urocitellus parryii* | 9999 | *Smcy,*  microsatellite | JQ361905,  NA | Y chromosome specific fragment | duplex PCR of male specific and control region | Gorrell et al. | 2012 | PMID: 22726203 |
| Yellow-bellied marmot | *Marmota flaviventris* | 93162 | *Smcy,*  microsatellite | JQ361907,  NA | Y chromosome specific fragment | duplex PCR of male specific and control region | Gorrell et al. | 2012 | PMID: 22726203 |
| Hoary marmot | *Marmota caligata* | 93160 | *Smcy,*  microsatellite | JQ361906,  NA | Y chromosome specific fragment | duplex PCR of male specific and control region | Gorrell et al. | 2012 | PMID: 22726203 |
| Woodchuck | *Marmota monax* | 9995 | *Smcy,*  microsatellite | JQ361908,  NA | Y chromosome specific fragment | duplex PCR of male specific and control region | Gorrell et al. | 2012 | PMID: 22726203 |
| Mouse | *Mus musculus,* CD1 | 10088 | Sry ,  Myog | 21674,  17928 | Y chromosome specific fragment | duplex PCR of male specific and control region | [Yano](https://www.ncbi.nlm.nih.gov/pubmed/?term=Yano%20T%5BAuthor%5D&cauthor=true&cauthor_uid=8260720) | 1993 | PMDI: 8260720 |
| Mouse | *Mus musculus,* 129S7/SvEvBrdBkl-*Hprt*^b-m2^_, (_129P3/J, BALB/cByJ, CBA/CaJ, DBA/2J, FVB/NJ, *Mus musculus* molossinus JF1/Ms, *Mus spretus* SPRET/EiJ | 10088 | *Kdm5d (Jarid1d)*,  *Kdm5c (Jarid1c)* | 20592,  20591 | indel | simplex PCR | [Clapcote and Roder](https://www.ncbi.nlm.nih.gov/pubmed/?term=Clapcote%20SJ%5BAuthor%5D&cauthor=true&cauthor_uid=15945368) | 2005 | PMID: 15945368 |
| Mouse | *Mus musculus,* CD1, Quackenbush, C57BL/6, Balb/c, FVB, 129, CBA.C57BL/6 | 10088 | *Sly,*  *Xlr* | 382301,  22441 | indel | simplex PCR | McFarlane et al. | 2013 | PMID: 23571295 |
| Mouse | *Mus musculus*,  CF1× C57BL/6J | 10088 | *Jarid1d,*  *Jarid1c* | 20592,  20591 | indel | simplex qPCR | [Prantner et al.](https://www.ncbi.nlm.nih.gov/pubmed/?term=Prantner%20AM%5BAuthor%5D&cauthor=true&cauthor_uid=26541770) | 2016 | PMID: 26541770 |
| Mouse | *Mus musculus,* CF1× C57BL/6J | 10088 | *Sry,*  *Il2* | 6736,  3558 | Y chromosome specific fragment | duplex PCR | [Prantner et al.](https://www.ncbi.nlm.nih.gov/pubmed/?term=Prantner%20AM%5BAuthor%5D&cauthor=true&cauthor_uid=26541770) | 2016 | PMID: 26541770 |
| Human | *Homo sapiens* | 9606 | *DYZ1* (pHY10 repeats, B fragment - male specific),  *AMELY*  *AMELX* | NA,  266,  265 | Y chromosome specific fragment, indel | PCR, AMEL simplex | Akane et al. | 1991 | PMID: 2032670 |
| Human | *Homo sapiens* | 9606 | *AMELY,*  *AMELX* | 266,  265 | indel | PCR | Sullivan et al. | 1993 | PMID: 8251166 |
| Human | *Homo sapiens* | 9606 | DXYS156X ,  DXYS156Y | NA | X and Y specific number of repeats | PCR | Chen et al. | 1994 | PMID: 7833950 |
| Human | *Homo sapiens* | 9606 | *AMELY,*  *AMELX* | 266,  265 | indel | PCR | Haas-Rochholz | 1997 | PMID: 9387013 |
| Human | *Homo sapiens* | 9606 | SRY markers (DYS19, DYS389I, DYS389II, DYS390, DYS391, DYS392, DYS393 , DYS385),  *AMELY,*  *AMELX* | 6736,  266,  265 | Y chromosome specific fragment, indel | multiplex PCR | Steinlechner et al. | 2002 | PMID: 12056519 |
| Human | *Homo sapiens* | 9606 | *SRY,*  *TSPY1 (DYS14)* | 6736,  7258 | Y chromosome specific fragment | qPCR | Zimmermann et al. | 2005 | PMID: 16020496 |
| Human | *Homo sapiens* | 9606 | *AMELY,*  *AMELX* | 266,  265 | indel, SSSP | PCR | Kashyap et al. | 2006 | PMID: 16603093 |
| Human | *Homo sapiens* | 9606 | *AMELY,*  *AMELX* | 266,  265 | indel | PCR, pyrosequencing | Tschentscher | 2008 | PMID: 18351373 |
| Human | *Homo sapiens* | 9606 | *SRY*,  2 *AMELY* regions,  2 *AMELX* regions,  four mini X-STR loci | 6736,  266,  265,  NA | indel, Y chromosome specific fragment, female heterozygosity of repeats on X | multiplex (genderplex) | Codina et al. | 2009 | PMID: 19089439 |
| Human | *Homo sapiens* | 9606 | *AMELY,*  *AMELX* | 266,  265 | indel, SSSP | nested PCR | Gibbon et al. | 2009 | PMID: 19215875 |
| Human | *Homo sapiens* | 9606 | *SRY* | 6736 | Y chromosome specific fragment | PCR singleplex | Kastelic et al. | 2009 | PMID: 19302388 |
| Human | *Homo sapiens* | 9606 | *TSPY1 (DYS14),*  *GAPDH* | 7258,  2597 | Y chromosome specific fragment | qPCR | Blagodatskikh et al. | 2010 | WOS: 000280702100010 |
| Human | *Homo sapiens* | 9606 | *SRY*,  X chromosome microsatellite *DXZ4* | 6736,  NA | Y chromosome specific fragment | nested PCR | Luptakova et al. | 2011 | PMID: 20851541 |
| Human | *Homo sapiens* | 9606 | STS,  AMELY,  *AMELX*,  two sequences of SRY and  their homologous sequences on X and 7 chromosome | 412,  266,  265,  6736 (SRY),  NA, NA | Y chromosome specific fragment, indel | multiplex PCR | Morikawa et al. | 2011 | PMID: 21519369 |
| Human | *Homo sapiens* | 9606 | *AMELY,*  *AMELX* | 266,  265 | indel, SSSP | pyrosequencing | Li et al. | 2012 | PMID: 22311027 |
| Human | *Homo sapiens* | 9606 | *SRY ,*  *AMELY,*  *AMELX* | 6736,  266,  265 | Y chromosome specific fragment, indel | duplex PCR | Tozzo et al. | 2013 | PMID: 23756502 |
| Human | *Homo sapiens* | 9606 | DYZ5 (male),  Alu,  mtDNA 12S,  MT-CYB | NA,  NA,  NA,  4519 | Y chromosome specific fragment | qPCR | Benoit et al. | 2013 | PMID: 23155118 |
| Human | *Homo sapiens* | 9606 | *SRY,*  *TSPY1 (DYS14*, multicopy) | 6736,  7258 | Y chromosome specific fragment | duplex qPCR | Kamodyová et al. | 2013 | PMID: 22917815 |
| Human | *Homo sapiens* | 9606 | *TSPY4,*  *SRY,*  *AMELY,*  *AMELX* | 728395,  6736,  266,  265 | Y chromosome specific fragment, indel | multiplex PCR | Jacot et al. | 2013 | PMID: 23312930 |
| Human | *Homo sapiens* | 9606 | *TSPY7,*  *TSPY8,*  *TSPY1 (DYS14),*  *SRY,*  *HBB* | NA,  728403,  7258,  6736,  3043 | Y chromosome specific fragment, indel | nested PCR | Campos et al. | 2014 | PMID: 24792405 |
| Human | *Homo sapiens* | 9606 | X and Y specific sequences | NA | Y chromosome specific fragment | multiplex PCR-HRM | Madel et al. | 2016 | PMID: 27613970 |
| Human | *Homo sapiens* | 9606 | whole genome | NA | sex specific dose variation | shotgun sequencing and calculation of Ry and Rx from number of alignments to sex chromosomes | [Mittnik et al.](https://www.ncbi.nlm.nih.gov/pubmed/?term=Mittnik%20A%5BAuthor%5D&cauthor=true&cauthor_uid=27706187) | 2016 | PMID: 27706187 |
| Indian mongoose | *Herpestes auropunctatus* | 48418 | *EIF2S3Y,*  *EIF2S3X* | AB550793,  AB550794 | indel | PCR | Murata et al. | 2011 | PMID: 21429150 |
| Red panda | *Ailurus fulgens* | 9649 | *ZFY,*  *ZFX* | HM802258,  HM802257 | allele specific sequences | triple primer PCR identification of sex specific amplicon length variation due to primer placing (ARMS) | Li et al. | 2011 | PMID: 21429153 |
| Giant panda | *Ailuropoda melanoleuca* | 9646 | *SRY ,*  *ZFY,*  *ZFX* | AM748312,  AM748304,  AM941054 | Y chromosome specific fragment | duplex PCR of male specific and control region | Pages et al. | 2009 | WOS:000267104000009 |
| Giant panda | *Ailuropoda melanoleuca* | 9646 | *SMCY,*  *318.2,*  *ZFX* | AB261830.1,  NA,  AB261822 | Y chromosome specific fragment | double male specific positive PCR test | [Bidon et al.](https://www.ncbi.nlm.nih.gov/pubmed/?term=Bidon%20T%5BAuthor%5D&cauthor=true&cauthor_uid=23347586) | 2013 | PMID: 23347586 |
| Polar bear | *Ursus maritimus* | 29073 | *SRY,*  *ZFY,*  *ZFX* | AM748305,  AM748297,  AM941048 | Y chromosome specific fragment | duplex PCR of male specific and control region | Pages et al. | 2009 | WOS:000267104000009 |
| Polar bear | *Ursus maritimus* | 29073 | *SMCY,*  *318.2 ,*  *ZFX* | AB261824.1,  NA,  AB261816.1 | Y chromosome specific fragment | double male specific positive PCR test | [Bidon et al.](https://www.ncbi.nlm.nih.gov/pubmed/?term=Bidon%20T%5BAuthor%5D&cauthor=true&cauthor_uid=23347586) | 2013 | PMID: 23347586 |
| Brown bear | *Ursus arctos* | 9644 | *SRY,*  *ZFY,*  *ZFX* | AM748306,  AM748298,  AM941047 | Y chromosome specific fragment | duplex PCR of male specific and control region | Pages et al. | 2009 | WOS:000267104000009 |
| Brown bear | *Ursus arctos* | 9644 | ZFY,  ZFX, or  SRY,  mtDNA | AM748298,  AM941047,  AM748306,  NA | Y chromosome specific fragment | duplex PCR of male specific and control region | Pages et al. | 2009 | WOS:000267104000009 |
| Brown bear | *Ursus arctos* | 9644 | *SMCY,*  *318.2,*  *ZFX* | AB261823.1,  HF547901,  AB261815.1 | Y chromosome specific fragment | double male specific positive PCR test | [Bidon et al.](https://www.ncbi.nlm.nih.gov/pubmed/?term=Bidon%20T%5BAuthor%5D&cauthor=true&cauthor_uid=23347586) | 2013 | PMID: 23347586 |
| American black bear | *Ursus americanus* | 9643 | *SRY,*  *ZFY/X* | AM748307,  AM748299,  AM941049 | Y chromosome specific fragment | duplex PCR of male specific and control region | Pages et al. | 2009 | WOS:000267104000009 |
| American black bear | *Ursus americanus* | 9643 | *SMCY,*  318.2,  *ZFX* | AB261825.1,  NA,  AB261817.1 | Y chromosome specific fragment | double male specific positive PCR test | [Bidon et al.](https://www.ncbi.nlm.nih.gov/pubmed/?term=Bidon%20T%5BAuthor%5D&cauthor=true&cauthor_uid=23347586) | 2013 | PMID: 23347586 |
| Asiatic black bear | *Ursus thibetanus* | 9642 | *SRY,*  *ZFY,*  *ZFX* | AM748310,  AM748302,  AM941050 | Y chromosome specific fragment | duplex PCR of male specific and control region | Pages et al. | 2009 | WOS:000267104000009 |
| Asiatic black bear | *Ursus thibetanus* | 9642 | *SMCY*,  318.2,  *ZFX* | AB261826.1,  NA,  AB261818 | Y chromosome specific fragment | double male specific positive PCR test | [Bidon et al.](https://www.ncbi.nlm.nih.gov/pubmed/?term=Bidon%20T%5BAuthor%5D&cauthor=true&cauthor_uid=23347586) | 2013 | PMID: 23347586 |
| Spectacled Bear | *Tremarctos ornatus* | 9638 | *SRY,*  *ZFY,*  *ZFX* | AM748311, AM748303, AM941053 | Y chromosome specific fragment | duplex PCR of male specific and control region | Pages et al. | 2009 | WOS:000267104000009 |
| Spectacled bear | *Tremarctos ornatus* | 9638 | *SMCY,*  318.2*,*  *ZFX* | AB261829.1,  NA,  AB261821 | Y chromosome specific fragment | double male specific positive PCR test | [Bidon et al.](https://www.ncbi.nlm.nih.gov/pubmed/?term=Bidon%20T%5BAuthor%5D&cauthor=true&cauthor_uid=23347586) | 2013 | PMID: 23347586 |
| Sloth bear | *Melursus ursinus* | 9636 | *SRY,*  *ZFY,*  *ZFX* | AM748309,  AM748301,  AM941052 | Y chromosome specific fragment | duplex PCR of male specific and control region | Pages et al. | 2009 | WOS:000267104000009 |
| Sloth bear | *Melursus ursinus* | 9636 | *SMCY,*  318.2,  *ZFX* | AB261828.1,  NA,  AB261820 | Y chromosome specific fragment | double male specific positive PCR test | [Bidon et al.](https://www.ncbi.nlm.nih.gov/pubmed/?term=Bidon%20T%5BAuthor%5D&cauthor=true&cauthor_uid=23347586) | 2013 | PMID: 23347586 |
| Sun bear | *Helarctos malayanus* | 9634 | *AMELY (AMLY),*  *AMELX (AMLX)* | AM941067,  AM941061 | indel | PCR identification of sex specific length variation due to indels | Pages et al. | 2009 | WOS:000267104000009 |
| Sun bear | *Helarctos malayanus* | 9634 | *SRY ,*  *ZFY,*  *ZFX* | AM748308,  AM748300,  AM941051 | Y chromosome specific fragment | duplex PCR of male specific and control region | Pages et al. | 2009 | WOS:000267104000009 |
| Sun bear | *Helarctos malayanus* | 9634 | *SMCY,*  318.2*,*  *ZFX* | AB261827.1,  NA,  AB261819 | Y chromosome specific fragment | double male specific positive PCR test | [Bidon et al.](https://www.ncbi.nlm.nih.gov/pubmed/?term=Bidon%20T%5BAuthor%5D&cauthor=true&cauthor_uid=23347586) | 2013 | PMID: 23347586 |
| San Joaquin kit fox, (coyote, gray fox, red fox, dog, maned wolf) | *Vulpes macrotis mutica, (Canis latrans, Urocyon cinereoargenteus, Vulpes vulpes, Canis familiaris, Chrysocyon brachyurus)* | 9630 | *ZFY,*  *ZFX* | AY310920,  AY310919 | SSSP | PCR-RFLP | Ortega et al. | 2004 | WOS:000226093800011 |
| Dog | *Canis lupus familiaris* | 9615 | *SRY,*  *ACTB* | NA | Y chromosome specific fragment | PCR of male specific sequence with a control | [Prugnard et al.](https://www.ncbi.nlm.nih.gov/pubmed/?term=Prugnard%20C%5BAuthor%5D&cauthor=true&cauthor_uid=26748721) | 2016 | PMID: 26748721 |
| American mink | *Mustela/Neovison vison* | 452646 | *SRY* | NA | Y chromosome specific fragment | PCR of male specific sequence | Statham et al. | 2007 | WOS:000250416200008 |
| American mink | *Mustela/Neovison vison* | 452646 | *ZFY,*  *ZFX* | AM039485,  AM039484 | SSSP | PCR-RFLP | Statham et al. | 2007 | WOS:000250416200008 |
| American mink | *Mustela/Neovison vison* | 452646 | *ZFY,*  *ZFX* | AM039485,  AM039484 | SSSP | direct sequencing of PCR; double peaks | Statham et al. | 2007 | WOS:000250416200008 |
| American mink | *Mustela/Neovison vison* | 452646 | *ZFY,*  *ZFX* | AM039485,  AM039484 | indel | PCR identification of sex specific length variation due to indels | Statham et al. | 2007 | WOS:000250416200008 |
| Ermine or stoat | *Mustela erminea* | 36723 | *SRY* | NA | Y chromosome specific fragment | PCR of male specific sequence | Statham et al. | 2007 | WOS:000250416200008 |
| Ermine or stoat | *Mustela erminea* | 36723 | *ZFY,*  *ZFX* | AM039483,  AM039482 | indel | PCR identification of sex specific length variation due to indels | Statham et al. | 2007 | WOS:000250416200008 |
| Ermine or stoat | *Mustela erminea* | 36723 | *ZFY,*  *ZFX* | AM039483,  AM039482 | SSSP | PCR-RFLP | Statham et al. | 2007 | WOS:000250416200008 |
| Ermine or stoat | *Mustela erminea* | 36723 | *ZFY,*  *ZFX* | AM039483,  AM039482 | SSSP | direct sequencing of PCR; double peaks | Statham et al. | 2007 | WOS:000250416200008 |
| Siberian weasel | *Mustela sibirica* | 36240 | *DDX3Y,*  *DDX3X* | AB530253,  AB530252 | SSSP | nested PCR-RFLP | Sekiquchi et al. | 2010 | PMID: 21565122 |
| Siberian weasel | *Mustela sibirica* | 36240 | *DDX3Y,*  *DDX3X* | AB530253,  AB530252 | Y chromosome specific fragment | multiplex PCR of male specific and control region | Sekiquchi et al. | 2010 | PMID: 21565122 |
| Japanese weasel | *Mustela itatsi* | 36238 | *DDX3Y,*  *DDX3X* | AB530251,  AB530250 | SSSP | nested PCR-RFLP | Sekiquchi et al. | 2010 | PMID: 21565122 |
| Japanese weasel | *Mustela itatsi* | 36238 | *DDX3Y,*  *DDX3X* | AB530251,  AB530250 | Y chromosome specific fragment | multiplex PCR of male specific and control region | Sekiquchi et al. | 2010 | PMID: 21565122 |
| Japanese marten | *Martes melampus* | 36721 | *DDX3Y,*  *DDX3X* | AB530255,  AB530254 | indel | nested PCR | Sekiquchi et al. | 2010 | PMID: 21565122 |
| Japanese marten | *Martes melampus* | 36721 | *DDX3Y,*  *DDX3* | AB530255,  AB530254 | Y chromosome specific fragment | multiplex PCR of male specific and control region | Sekiquchi et al. | 2010 | PMID: 21565122 |
| European pine marten | *Martes martes* | 29065 | *ZFY,*  *ZFX* | AM039479,  AM039478 | indel | PCR identification of sex specific length variation due to indels | Statham et al. | 2007 | WOS:000250416200008 |
| European pine marten | *Martes martes* | 29065 | *SRY* | NA | Y chromosome specific fragment | PCR of male specific sequence | Statham et al. | 2007 | WOS:000250416200008 |
| European pine marten | *Martes martes* | 29065 | *ZFY,*  *ZFX* | AM039479,  AM039478 | SSSP | PCR-RFLP | Statham et al. | 2007 | WOS:000250416200008 |
| European pine marten | *Martes martes* | 29065 | *ZFY,*  *ZFX* | AM039479,  AM039478 | SSSP | direct sequencing of PCR; double peaks | Statham et al. | 2007 | WOS:000250416200008 |
| Eurasian badger | *Meles meles* | 9662 | *SRY* | NA | Y chromosome specific fragment | PCR of male specific sequence | Statham et al. | 2007 | WOS:000250416200008 |
| Eurasian badger | *Meles meles* | 9662 | *ZFY,*  *ZFX* | AM039481,  AM039480 | SSSP | PCR-RFLP | Statham et al. | 2007 | WOS:000250416200008 |
| Eurasian badger | *Meles meles* | 9662 | *ZFY,*  *ZFX* | AM039481,  AM039480 | SSSP | direct sequencing of PCR; double peaks | Statham et al. | 2007 | WOS:000250416200008 |
| Eurasian otter | *Lutra lutra* | 9657 | *ZFY,*  *ZFX* | AM039477,  AM039476 | indel | PCR identification of sex specific length variation due to indels | Statham et al. | 2007 | WOS:000250416200008 |
| Eurasian otter | *Lutra lutra* | 9657 | *SRY* | NA | Y chromosome specific fragment | PCR of male specific sequence | Statham et al. | 2007 | WOS:000250416200008 |
| Eurasian otter | *Lutra lutra* | 9657 | *ZFY,*  *ZFX* | AM039477, AM039476 | SSSP | PCR-RFLP | Statham et al. | 2007 | WOS:000250416200008 |
| Eurasian otter | *Lutra lutra* | 9657 | *ZFY,*  *ZFX* | AM039477, AM039476 | SSSP | direct sequencing of PCR; double peaks | Statham et al. | 2007 | WOS:000250416200008 |
| Euroasian otter | *Lutra lutra* | 9657 | *ZFY,*  *ZFX* | AB491597, AB491606 | Y chromosome specific fragment | qPCR with TaqMan probe | O'Neill et al. | 2013 | PMID: 23870402 |
| Eastern red bat | *Lasiurus borealis* | 258930 | *ZFY,*  *ZFX* | described in the article | Y chromosome specific fragment | duplex PCR of male specific and control region | Korstian et al. | 2013 | PMID: 23647806 |
| Hoary bat | *Lasiurus cinereus* | 257879 | *ZFY,*  *ZFX* | described in the article | Y chromosome specific fragment | duplex PCR of male specific and control region | Korstian et al. | 2013 | PMID: 23647806 |
| Tri-coloured bat | *Pipistrellus subflavus* | 27672 | *ZFY,*  *ZFX* | described in the article | Y chromosome specific fragment | duplex PCR of male specific and control region | Korstian et al. | 2013 | PMID: 23647806 |
| Evening bat | *Nycticeius humeralis* | 27670 | *ZFY,*  *ZFX* | described in the article | Y chromosome specific fragment | duplex PCR of male specific and control region | Korstian et al. | 2013 | PMID: 23647806 |
| Silver-haired bat | *Lasionycteris noctivagans* | 27667 | *ZFY,*  *ZFX* | described in the article | Y chromosome specific fragment | duplex PCR of male specific and control region | Korstian et al. | 2013 | PMID: 23647806 |
| Mexican free-tailed bat | *Tadarida brasiliensis* | 9438 | *ZFY,*  *ZFX* | described in the article | Y chromosome specific fragment | duplex PCR of male specific and control region | Korstian et al. | 2013 | PMID: 23647806 |
| Blue sheep | *Pseudois nayaur szechuanensis* | 1204301 | *AMELY,*  *AMELX* | NA | indel | PCR identification of sex specific length variation due to indels | [Liu et al.](https://www.ncbi.nlm.nih.gov/pubmed/?term=Liu%20X%5BAuthor%5D&cauthor=true&cauthor_uid=26345836) | 2015 | PMID: 26345836 |
| Blue sheep | *Pseudois nayaur nayaur* | 1204301 | *AMELY,*  *AMELX* | NA | indel | PCR identification of sex specific length variation due to indels | [Liu et al.](https://www.ncbi.nlm.nih.gov/pubmed/?term=Liu%20X%5BAuthor%5D&cauthor=true&cauthor_uid=26345836) | 2015 | PMID: 26345836 |
| Sheep | *Ovis aries* | 9940 | *UcdO43 ,*  *ZFY,*  *ZFX* | U65982,  NA,  101114932 | Y chromosome specific fragment, SSSP | nested PCR-RFLP | [Gutiérrez-Adán et al.](https://www.ncbi.nlm.nih.gov/pubmed/?term=Guti%C3%A9rrez-Ad%C3%A1n%20A%5BAuthor%5D&cauthor=true&cauthor_uid=9172311) | 1997 | PMID: 9172311 |
| Sheep | *Ovis aries* | 9940 | *ZFY,*  *ZFX* | NA,  101114932 | SSSP | PCR-RFLP | Saravanan et al. | 2003 | WOS:000181916600004 |
| Sheep | *Ovis aries* | 9940 | *SRY*,  1.715 satellite | 100529253,  NA | Y chromosome specific fragment | duplex PCR of male specific and control region | [Mara et al.](https://www.ncbi.nlm.nih.gov/pubmed/?term=Mara%20L%5BAuthor%5D&cauthor=true&cauthor_uid=15278902) | 2004 | PMID: 15278902 |
| Sheep | *Ovis aries* | 9940 | *HMG* box,  *ACTB* | 100529253 (SRY),  100885765 | Y chromosome specific fragment | duplex PCR of male specific and control region | [Salabi et al.](https://www.ncbi.nlm.nih.gov/pubmed/?term=Salabi%20F%5BAuthor%5D&cauthor=true&cauthor_uid=25984504) | 2014 | PMID: 25984504 |
| Sheep | *Ovis aries* | 9940 | *AMELY,*  *AMELX* | 448815,  101118759 | indel | PCR identification of sex specific length variation due to indels | [Tavares et al.](https://www.ncbi.nlm.nih.gov/pubmed/?term=Tavares%20KC%5BAuthor%5D&cauthor=true&cauthor_uid=27050974) | 2016 | PMID: 27050974 |
| Goat | *Capra hircus* | 9925 | *AMELY,*  *AMELX* | 108634548,  100860759 | allele specific sequences | triple primer PCR identification of sex specific amplicon length variation due to primer placing (ARMS) | [Tsai et al.](https://www.ncbi.nlm.nih.gov/pubmed/?term=Tsai%20TC%5BAuthor%5D&cauthor=true&cauthor_uid=21421829) | 2011 | PMID: 21421829 |
| Watter buffalo | *Bubalus bubalis* | 89462 | *ZFY,*  *ZFX* | NA,  102395265 | SSSP | PCR-RFLP | [Pande and Totey](https://www.ncbi.nlm.nih.gov/pubmed/?term=Pande%20A%5BAuthor%5D&cauthor=true&cauthor_uid=9526699) | 1998 | PMID: 9526699 |
| Watter buffalo | *Bubalus bubalis* | 89462 | *BuRYN.I,*  *ZFY,*  *ZFX* | X93551.1,  NA,  102395265 | Y chromosome specific fragment, indel | male specific sequence amplified with nested primers in multiplex reaction with IPC | [Appa Rao and Totey](https://www.ncbi.nlm.nih.gov/pubmed/?term=Appa%20Rao%20KB%5BAuthor%5D&cauthor=true&cauthor_uid=10729003) | 1999 | PMID: 10729003 |
| Watter (swamp and river) buffalo | *Bubalus bubalis* | 89462 | *BuRY.2*,  12S rRNA | M74507,  102396597 or AJ457159 | Y chromosome specific fragment | LAMP reaction of male specific sequence and control LAMP of male-female common sequence | [Hirayama et al.](https://www.ncbi.nlm.nih.gov/pubmed/?term=Hirayama%20H%5BAuthor%5D&cauthor=true&cauthor_uid=16672158) | 2006 | PMID: 16672158 |
| Swamp buffalo | *Bubalus bubalis* | 89462 | *SRY* (*HMG* box),  *G3PDH* | 102411616,  102404028 | Y chromosome specific fragment | nested duplex PCR of male specific sequence and control | [Fu et al.](https://www.ncbi.nlm.nih.gov/pubmed/?term=Fu%20Q%5BAuthor%5D&cauthor=true&cauthor_uid=17928043) | 2007 | PMID: 17928043 |
| cattle | *Bos taurus* | 9913 | *FBNY,*  *FBN17* | AJ002548,  253604 | Y chromosome specific fragment | simplex PCR | [Weikard et al.](https://www.ncbi.nlm.nih.gov/pubmed/?term=Weikard%20R%5BAuthor%5D&cauthor=true&cauthor_uid=11550263) | 2001 | PMID: 11550263 |
| cattle | *Bos taurus* | 9913 | S4,  1.715 | NA | Y chromosome specific fragment | LAMP reaction of male specific sequence and control LAMP of male-female common sequence | Hirayama et al. | 2004 | PMID: 15251240 |
| cattle | *Bos taurus* | 9913 | *SRY,*  *AMELY,*  *AMELX* | 280931,  281621,  281620 | Y chromosome specific fragment, indel | Duplex PCR of male specific and control region | Gokulakrishnan et al. | 2012 | WOS:000301847300005 |
| cattle | *Bos taurus* | 9913 | *AMELY,*  *AMELX* | 281621,  281620 | indel | PCR identification of sex specific length variation due to indels | [Tavares et al.](https://www.ncbi.nlm.nih.gov/pubmed/?term=Tavares%20KC%5BAuthor%5D&cauthor=true&cauthor_uid=27050974) | 2016 | PMID: 27050974 |

NA – data not available or not applicable; ARMS – amplification refractory mutation system; IPC – internal positive control; ID – identification number; SSSP – sex specific sequence polymorphisms; SSSV – sex sequence variant; LAMP – loop mediated isothermal amplification; HRM – high resolution melting curve; PCR – polymerase chain reaction; qPCR – quantitative PCR; RFLP – restriction fragment length polymorphism, WOS – Web of science ID; PMID – PubMed ID.
